# Dynamics of Oddball Sound Processing: Trial-by-Trial Modeling of ECoG Signals

**DOI:** 10.3389/fnhum.2021.794654

**Published:** 2022-02-10

**Authors:** Françoise Lecaignard, Raphaëlle Bertrand, Peter Brunner, Anne Caclin, Gerwin Schalk, Jérémie Mattout

**Affiliations:** ^1^Lyon Neuroscience Research Center, CRNL, INSERM, U1028, CNRS, UMR 5292, Lyon, France; ^2^University Lyon 1, Lyon, France; ^3^Department of Neurosurgery, Washington University School of Medicine, St. Louis, MO, United States; ^4^Department of Neurology, Albany Medical College, Albany, NY, United States; ^5^National Center for Adaptive Neurotechnologies, Albany, NY, United States

**Keywords:** single-trial analysis, predictive coding, mismatch negativity, Bayesian learning, general linear model, Bayesian model reduction

## Abstract

Recent computational models of perception conceptualize auditory oddball responses as signatures of a (Bayesian) learning process, in line with the influential view of the mismatch negativity (MMN) as a prediction error signal. Novel MMN experimental paradigms have put an emphasis on neurophysiological effects of manipulating regularity and predictability in sound sequences. This raises the question of the contextual adaptation of the learning process itself, which on the computational side speaks to the mechanisms of gain-modulated (or precision-weighted) prediction error. In this study using electrocorticographic (ECoG) signals, we manipulated the predictability of oddball sound sequences with two objectives: (i) Uncovering the computational process underlying trial-by-trial variations of the cortical responses. The fluctuations between trials, generally ignored by approaches based on averaged evoked responses, should reflect the learning involved. We used a general linear model (GLM) and Bayesian Model Reduction (BMR) to assess the respective contributions of experimental manipulations and learning mechanisms under probabilistic assumptions. (ii) To validate and expand on previous findings regarding the effect of changes in predictability using simultaneous EEG-MEG recordings. Our trial-by-trial analysis revealed only a few stimulus-responsive sensors but the measured effects appear to be consistent over subjects in both time and space. In time, they occur at the typical latency of the MMN (between 100 and 250 ms post-stimulus). In space, we found a dissociation between time-independent effects in more anterior temporal locations and time-dependent (learning) effects in more posterior locations. However, we could not observe any clear and reliable effect of our manipulation of predictability modulation onto the above learning process. Overall, these findings clearly demonstrate the potential of trial-to-trial modeling to unravel perceptual learning processes and their neurophysiological counterparts.

## Introduction

Recent computational models of perception address sound processing in oddball paradigms as the learning of regularities that pertain to the repetition of an acoustic pattern (typically a single tone in the basic form of oddball sequences, i.e., the *standard* stimuli). The corollary that follows is then to view mismatch responses elicited by unexpected *deviant* sounds as indexing surprise processing. In particular, the Mismatch Negativity (MMN; Näätänen et al., 1978) has been suggested to reflect a prediction error (Friston, 2005). This model of the MMN leverages on complex underlying (Bayesian) computations that raise the practical question of their neuronal implementation (Knill and Pouget, 2004). Deciphering these processes is a topic of intense research, both at the physiological (Garrido et al., 2009b; Auksztulewicz and Friston, 2016; Carbajal and Malmierca, 2018) and cognitive (Winkler, 2007; Heilbron and Chait, 2018) levels.

An important aspect that computational models of perception have put forward is the influence of the acoustic context onto sound processing in oddball paradigms, that is, as we shall see, explicitly formalized in popular predictive coding implementation (Friston, 2005; Spratling, 2016). Interestingly, this is in line with recent MMN findings which emphasized the importance of the ordering of experimental conditions (Fitzgerald and Todd, 2020; Todd et al., 2021), pointing out the need for refining our understanding of mismatch responses.

Surprise or prediction errors play a key role in perceptual inference and learning (Friston, 2009). Importantly, they are thought to drive belief updating in a context dependent manner. In other words, the context determines the relevance of a given prediction error, and whether it should be filtered out or accounted for by promoting some adaptation (Adams et al., 2013; Mathys et al., 2014). In the Bayesian framework, this context dependent modulation naturally emerges in the form of a precision weight. And surprise takes the more refined form of a precision-weighted prediction error (Bastos et al., 2012). Should the precision or confidence be low (e.g., in a noisy environment), the learning triggered by a new sound should be lessened to avoid irrelevant updates of the internal model. On the contrary, a high precision will amplify the prediction error, and yield a larger belief update.

The precision weighting account of contextual influences has led to manipulations of the statistical structure of oddball sequences to test specific predictions about the ensuing modulations of the MMN. In Garrido et al. (2013), auditory stimulations were sampled from a Gaussian distribution; larger amplitudes were measured at the MMN latency in response to outlier sounds when the distribution variance was reduced. This finding speaks to the precision of standard prediction, an aspect that has also been investigated recently using a different manipulation (SanMiguel et al., 2021). In this study, sound sequence comprised multiple tones occurring randomly, with two of them playing the role of deviants and standards, respectively. The proportion of standards in the sequence was manipulated while keeping deviant probability constant and larger deviant response at the MMN latency was reported in the more stable condition where standards were more frequent. Several studies also manipulated the predictability of sound sequences (Chennu et al., 2013; Recasens et al., 2014; Auksztulewicz and Friston, 2015; Lecaignard et al., 2015; Dürschmid et al., 2016; Auksztulewicz et al., 2017, 2018). In short, predictability has been associated with reduced brain responses, in line with expected smaller precision-weighted prediction errors (Lecaignard et al., 2015). However, enhanced brain activity has also been reported in predictability conditions (Barascud et al., 2016; Southwell et al., 2017). Together, these reports call for further investigations to shed light onto the computational mechanisms at play and their neurophysiological underpinnings. The current electrocorticographic (ECoG) study was intended to contribute to this effort.

Specifically, a closer look at the putative effect of predictability provides a plausible explanation for the above apparent contradictory findings. Indeed, predictability has a two-fold and opposite effect on prediction error and its precision, respectively. It is expected to decrease the former (as an unsurprising environment contributes to a more accurate prediction of future sensations) but to increase the latter (as a structured context provides more reliable prediction errors). An important aspect is that both computational variables depend upon the temporal structure of the sensory input sequence, but precision weight (or inverse variance) pertaining to second order statistics (in contrast with prediction pertaining to first-order ones) is expected to be optimized over a slower timescale (Mathys et al., 2014). As a consequence, averaging methods like traditional event-related potential (ERP) approaches will likely be unable to reveal the contribution of their respective dynamics onto brain activity, even if these dynamics become separable due to a predictability manipulation. To circumvent this issue, attempts have been made that consisted of comparing the mismatch responses obtained in the beginning and end of oddball sequences (Fitzgerald and Todd, 2018; Todd et al., 2021).

Alternatively, trial-by-trial analysis, pertaining to the examination of the single-trial activity elicited by single sounds, enables the direct examination of such dynamics. In a previous study using simultaneous EEG and MEG recordings, we coupled a predictability manipulation of an oddball paradigm with a single-trial data modeling approach (Lecaignard et al., 2021b). Trial-by-trial activity was found to be best predicted by a Bayesian learning model of the deviant probability and this model revealed a modulation of learning by sequence predictability, suggesting an automatic adaptation of sensory processing to the statistical structure of the auditory stream. This adaptation could be captured by a model parameter that determines the influence of past experience onto perceptual inference. The larger value we found under predictability can be interpreted as a larger memory span that fits well with the fact that the more structured the sound sequence, the more past information is integrated to make predictions These findings and few others speak to the plausibility of perceptual models engaged in oddball processing and the trial-by-trial fluctuations they prescribe (Ostwald et al., 2012; Lieder et al., 2013; Stefanics et al., 2014; Meyniel et al., 2016; Sedley et al., 2016; Weber et al., 2020).

We designed the present ECoG study around two objectives: first, we aimed at testing the reproducibility of the recent EEG-MEG single-trial findings, considering the youth of this field of research, and the methodological challenge on which it is based, i.e., the sensitivity of single-trial data to noise. We here expect ECoG data to refine the spatio-temporal characterization of perceptual learning because of its excellent spatial and temporal resolution. Second, to refine the description of cognitive process(es) engaged during the passive processing of sound, we propose a novel approach combining a general linear model (GLM) with advanced Bayesian methods for model comparison (Bayesian model reduction, Friston et al., 2016, 2018) to compare a learning regressor with non-learning ones. Using a GLM approach, competing cognitive hypotheses are no longer tested as mutually exclusive (as was the case in our prior EEG-MEG study) and we could examine where and when their related regressor each contributes to the observed data in a flexible way. It is interesting to note that this investigation, because it involves both dynamic and static models (learning and non-learning, respectively) also amounts to addressing the potential of the still little used single-trial modeling. In short, if dynamic models were found unlikely based on current data, single-trial modeling would appear too complex to reveal constant effects for which averaging methods like evoked potential analysis are perfectly relevant. Analysis of data from four implanted patients with ECoG electrodes over the temporal lobe provides substantial evidence for Bayesian learning in the brain and promotes single-trial modeling to further characterize auditory processing in the light of perceptual inference and predictive coding. Surprisingly, no clear evidence for the expected adaptation of learning under predictability could be disclosed.

## Materials and Methods

### Participants

Six patients (P1, P2, P3, P4, P5, and P6) with pharmacologically intractable epilepsy participated in this study at Albany Medical Center (Albany, NY, United States). They underwent pre-surgical monitoring with temporary placement of electrocorticographic grids over frontal, parietal, and temporal cortices. Four of the six patients (P2, P4, P5, and P6) were also assessed with intracranial depth electrodes located over temporal regions; analysis of the related data is not included in the present study. All patients provided informed consent for participating in the study, which was approved by the Institutional Review Board of Albany Medical College and the Human Research Protections Office of the United States Army Medical Research and Materiel Command. Table 1 summarizes the patients’ clinical profiles. Cortical views with electrode overlay are provided in Supplementary Figure S1.

**TABLE 1:** Clinical profiles of participants.

Subject	Age	Sex	Seizure focus	#grids	#strips	#electrodes
P1	69	M	Right temporal	1	11	92
P2	33	M	Left temporal	1	5	224
P3	51	M	Left temporal	1	6	126
P4	36	F	Right temporal	1	8	92
P5	27	F	Left temporal	2	4	93
P6	31	M	Left temporal	2	10	122

*The number of electrodes refers to contacts included in the current analysis (distant from epileptogenic foci, without electrical or mechanical artifacts).*

As explained in the experimental procedure section below, each patient received auditory stimuli divided into four runs during a single session. Two patients however followed a different scheme: patient P1 underwent two sessions (day 2 and day 5 after surgery) as well as patient P6 who received two runs in a first session (day 1) and six runs in a second one (day 2). Given that they did not report having noticed the statistical manipulation of the sound sequences (see below), and in order to take advantage of most of these data, we included them all in our subsequent analyses (we hereafter refer to these datasets by P1a, P1b, P6a and P6b, respectively). However, no data from patient P1 or from P6a survived our selection criterion (see below). Hence, only session P6b was included for subsequent analysis. For full transparency, we included the analysis of P6a as Supplementary Material (Supplementary Figure S2). Regarding patient P3, our analyses identified only one responsive location; we decided not to include the data in the study, and provide the related findings in Supplementary Material (Supplementary Figure S3). In summary, the present work relies on four datasets: P2, P4, P5, and P6b.

### Recordings

Implanted subdural grids (from PMT Corp., Chanhassen, MN, United States) were approved for human use and consisted of platinum-iridium electrodes (4 mm diameter, 2.4 mm exposed) that were embedded in silicone and spaced 6–10 mm from each other in five patients (P1, P3, P4, P5, and P6) and 3 mm in subject P2. Reference and ground were subdural electrodes distant from the epileptogenic area. Grid placement and duration of ECoG monitoring were determined to meet the requirements of the clinical evaluation.

Recordings were conducted at the patient bedside using BCI2000 (Schalk et al., 2004; Schalk and Mellinger, 2010^1^). Electrocorticographic signals were amplified using a 256-channel g.HIamp biosignal acquisition device (g.tec, Graz, Austria) and digitized at a sampling rate of 1200 Hz.

Sensor co-registration with cortical anatomy involved pre-operative magnetic resonance imaging (MRI) scans and post-operative computed tomography images (CT; Kubanek and Schalk, 2015), and was achieved using SPM8^2^. Supplementary Figure S1 shows for each patient the resulting estimates of 3D stereotactic coordinates overlaying cortical brain mesh extracted from individual MRI scans using FreeSurfer^3^.

### Experimental Procedure

Each patient underwent one recording session with four runs, except for patients P1 and P6 who received two sessions separated by 3 days (four runs each) and 1 day (two and six runs), respectively.

**FIGURE 1:**
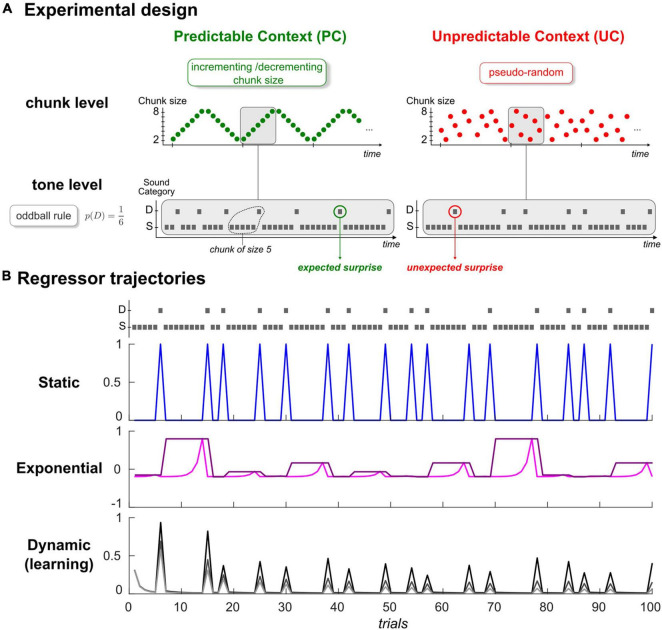
**(A)** Experimental Design. Schematic view of the predictability manipulation (chunk level) applying to typical oddball sound sequences (tone level). Predictable context (left, green) involves cycles of ordered transitions between segments of repeating standards (chunks), which become shuffled in the Unpredictable context (right, red). Average deviant probability remains the same in both contexts (*p* = 1/6). Gray rectangles delineate an exemplary cycle for both sequences. S: Standard, D: Deviant. **(B)** Examples of regressors for the static (top), exponential (middle), and dynamic (bottom) categories. Each trajectory was simulated with the first 100 tones of one subject in context UC, depicted in the upper panel following the standard/deviant representation in panel **(A)**. In the static category, deviant regressor is shown in blue (standard regressor is not displayed for convenience, as it mirrors deviant regressor). Exponential rank and chunk size regressors are presented in pink and purple, respectively. Regarding the learning regressor, three examples of Bayesian Surprise trajectories are provided, and were generated from different time constant values (parameter τ in (Eq. 3); 10,20 and 50: from dark to light gray).

Brain activity was recorded during an auditory oddball paradigm originally developed by our group (Lecaignard et al., 2015) and slightly modified here (see Figure 1A). Participants were instructed to ignore the sounds and watch a silent movie of their choice with subtitles. Each session lasted ∼50 min, including short breaks between runs. In the previous EEG-MEG study (Lecaignard et al., 2015), subjects were asked at the end of the experiment, to report to which extent they had been following the instruction to ignore the sounds and whether they had noticed the different sound attributes. Here, given the constraints related to the patients’ condition and the acquisitions conducted in a clinical context, these verifications were validated orally but we could not have an precise description of the participants’ sensory experience.

Auditory sequence in every run consisted of sounds (70 ms duration, 500 ms interstimulus interval) with repeating *standard* (500 Hz or 550 Hz) and unexpected frequency *deviants* (550 Hz or 500 Hz, occurrence probability *p* = 1/6). As shown in Figure 1A, in the predictable context (PC), deviants were delivered according to an incrementing-decrementing rule applied to the size of repeating standard segments (or chunks) while they were pseudo-randomly distributed among standards in the unpredictable context (UC). We considered specific controls for the number of standards between two deviants in context UC to ensure that despite their differing statistical structure, both sequence types (UC, PC) had the same deviant probability and the same distribution of chunk size (varying from 2 to 8 standards). Each context (UC, PC) was delivered in two runs to enable reversing the role of the two sounds (500 Hz/550 Hz; standard/deviant). Further details about stimuli and sequences can be found in Lecaignard et al. (2015). We used BCI2000 to deliver the acoustic stimuli that were presented binaurally through headphones.

### Data Processing

We used the MNE software for electrophysiological analysis (Gramfort et al., 2013) for raw data conversion to BIDS format^4^ and data preprocessing. Continuous recordings were band-pass filtered using a zero-phase finite impulse response (FIR) filter with Hann window in the 0.5–100 Hz band, notch-filtered at 60 Hz, 120 Hz, 180 Hz, and 240 Hz using a zero-phase FIR notch filter (stop band width at each frequency = 6 Hz) to remove the power line harmonics artifacts, and downsampled to 400 Hz. We excluded electrodes close to epileptogenic zones or electrodes whose ECoG signals were clearly artifactual based on visual inspection of the power spectral density. Time segments with obvious noise from electrical, mechanical or muscular origin were also rejected. Electrocorticographic recordings were referenced to the common averaged reference (CAR). We then extracted 600-ms-long epochs around the onset of the auditory stimuli (-100 to 500 ms around stimulus onset). Trial rejection was based on a peak-to-peak (maximum–minimum amplitude within epochs) threshold procedure applied to ECoG data: for each subject (except P2, see below), we first calculated for each location the distribution of peak-to-peak amplitudes over epochs. Next, at the level of the group of locations, we calculated the global distribution of mean values as well as that of outliers (two standard deviations from the mean). We rejected locations if their mean was found outlying the global mean (we call them as *bad* sensors). For the remaining locations, we used the outlier amplitude of the global outlier distribution as the threshold above which data segments were next rejected. The overall approach yielded the following threshold and rejection percentage: (364 uV; 26%), (330 uV; 19%), and (722 uV; 21%) for patients P4, P5, and P6b, respectively. In patient P2, data were contaminated by a lot of spikes; hence, we applied a threshold of 500 uV and obtained 39% of trial rejection. Regarding the datasets that were excluded, we obtained (586 uV; 16%), (461 uV; 16%), (742 uV; 13%), and (552 uV; 18%) in P1a, P1b, P3 and P6b, respectively. CAR referencing applied to the resulting sensor set.

We next applied a 2–20 Hz pass-band filter (zero-phase FIR) to continuous data, downsampled to 200 Hz for data reduction purpose and extracted the 600-ms epochs around the accepted trials resulting from the above-mentioned procedure. Finally, artifact-free and baseline-corrected epochs ([–100 +500] ms corresponding to *N*_*s*_ = 120 time samples) were exported into SPM12^2^.

### Rationale of the Modeling Approach

**FIGURE 2:**
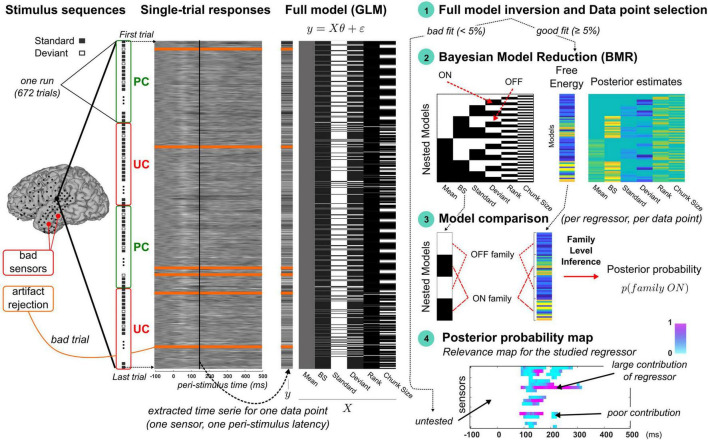
Overview of the trial-by-trial modeling approach. From **left** to **right**. **Stimulus sequences:** Four illustrative runs of an experimental session are represented vertically with standard and deviant indicated by gray and white squares, respectively, and UC and PC contexts delineated by red and green rectangles, respectively. Electrocorticographic sensor overlay on a brain cortical surface (from participant P5) indicates the sensors classified as bad (red) and those retained for the modeling (black). **Single-trial responses:** 2D map of single-trial activity (uV) measured at the highlighted sensor on the brain surface (large black dot). Each row represents the 600 ms epoch of signal elicited by a stimulus of the oddball sequence (first and last trials are shown with dotted arrows). Bad trials (orange lines) are included in the modeling as trial order matters in dynamic processes (the related noisy signal is not accounted for). **Full model (GLM):** an example of design matrix (X) is provided (right) showing typical regressor trajectories (columns) over trials (rows). Data to be fitted (y) pertains to the trial-by-trial time series extracted at a particular latency (vertical black line overlaying the 2D single-activity map). **Step 1, Full model fitting and selection of data points:** data point is found responsive to the full model if goodness of fit is larger than 5%. In this case, the model comparison procedure (steps 2 to 4) is performed. **Step 2, Bayesian model reduction:** left panel (model space): each model (row) corresponds to a nested version of the full model obtained by switching ON (black) and OFF (white) the different regressors. Bottom row represents the full model (all regressors are ON). Right panel: BMR is applied to each nested model to derive the free energy and posterior estimates of model parameters. **Step 3, Model comparison:** for each regressor, two families are compared using family-level inference (Penny et al., 2010). The ON family (indicated here for the dynamic regressor, with BS denoting the Bayesian Surprise) includes all the ON models (black) with associated free energies as indicated with the dotted red lines. The OFF family is defined similarly based on the OFF models (white). **Step 4, Posterior probability map:** Posterior probability of the ON family is then computed at each responsive data point to derive a spatio-temporal map of the regressor’s relevance. At each sensor (*y*-axis) and each peri-stimulus latency (*x*-axis), color intensity (from blue to pink) reflect the posterior probability (in [0, 1]). White points corresponds to untested data points due to an unreliable fitting of the full model (unresponsive data point). All maps in this figure are shown with arbitrary color scales.

In order to test Bayesian learning as the perceptual model the brain would use when exposed to oddball sounds, as well as its automatic modulation under predictability (as we found using EEG-MEG), we considered a modeling framework based on advanced Bayesian methods and applying to single-trial activity. We here introduce to the overall procedure, which is depicted in Figure 2.

Single-trial activity here corresponds to the signals measured at ECoG sensors and induced by the presentation of a single stimulus. Single-trial signals are naturally relevant to investigate the functional interpretation of trial-by-trial fluctuations, that should reflect the updates of computational (learning) quantities if the brain was to entertain such learning. In this paper, the notion of dynamics refers to the temporal dependencies that take place over the time-course of the experiment (meaning that trial order matters). Considering that time-dependent influence is also critical for learning, we refer to dynamic or learning process equivalently. Dynamic processes differ from static ones where in this case past experience is not accounted for in stimulus processing. It should be noticed that the dynamic-based examination of brain activity is not possible using typical evoked responses, as the averaging of single-trial content is precisely meant to get rid of the dynamic information. In the following, we will refer to trial-by-trial dynamics (or trajectory) as the time series extracted over one or multiple experimental run(s) at a particular sensor and a particular peri-stimulus sample. And we will call data point the spatio-temporal location where it is measured (one sensor, one peri-stimulus sample). An example is illustrated in Figure 2 (panel “Single-trial responses”). In the present work, there were *N_t_* = 672 single trials per run, that each involves a 600 ms temporal window (*N_s_* = 120 peri-stimulus samples). In total, for each participant, *N*_*c*_×*N*_*s*_ trial-by-trial time series contributed to the present findings, with *N_c_* the number of good channels (retained after artifact rejection).

The above-cited trial-by-trial modeling studies that have been conducted using oddball paradigms confronted brain signals (single-trial dynamics) with several model predictions that each reflected a possible account of sound processing (Ostwald et al., 2012; Lieder et al., 2013; Stefanics et al., 2014; Meyniel et al., 2016; Sedley et al., 2016; Weber et al., 2020; Lecaignard et al., 2021b). Typically, each model was treated separately and Bayesian model comparison (Penny et al., 2010) was then employed to select which one was more likely to have generate the observed data. Here, we considered a different approach based on a GLM in order to evaluate the contribution of each cognitive account to the data, in a way that does not preclude a mixture of several ones (thanks to the linear combination). We expected this scheme, because it is more flexible, to provide a finer spatio-temporal description of the mechanisms underlying oddball sound processing.

As can be seen in Figure 2 and as will be described in Section “Statistical Model,” we first considered a GLM comprising six different regressors (each detailed below) and that we call the “full model.” For each participant (P2, P4, P5 and P6b), it was fitted to the trial-by-trial activity extracted at each data point (defined in space and time at all good sensors and all peri-stimulus samples). If the resulting goodness of fit was acceptable (according to a criterion described in Section “Data Point Selection”), the data point was considered as model responsive and included for subsequent analysis. The latter aims at identifying the regressor(s) responsible for such responsiveness and rests on steps 2 to 4 of our methodological framework depicted in Figure 2 (right panel). In step 2, we considered alternative models of the full one, obtained by switching ON and OFF the contributions of all regressors; we employed Bayesian Model Reduction (BMR; Friston et al., 2016) to derive efficiently specific Bayesian quantities that are necessary for the model comparison that comes next. Precisely, model comparison (step 3) was then conducted for each regressor independently at the level of families of models, grouping models where the regressor of interest is present (we will refer to the ON family) and models where it is not (the OFF family). Using family-level inference (Penny et al., 2010), we obtained the posterior probability of the ON family which quantifies how likely the regressor is to have contributed to the data (note that the sum of the ON and OFF family posterior probabilities is equal to 1). Applying this scheme (step 1 to step 3) to all data points (step 4) yields a spatio-temporal description of the regressor’s relevance. Such posterior probability map (referring to the ON family) could be computed for each regressor. For sake of clarity, the notion of model will now refer to the GLMs (the full or nested variants) and we will use the term “regressor” to mention the different accounts of sensory processing that we test (some of them were tested as separate “models” in the above-mentioned trial-by-trial studies). This terminology emphasizes the fact that current alternative accounts are not tested as competing in this work.

This modeling approach was first applied to data in both contexts (UC and PC, four runs) to examine sensory processing and test it as Bayesian learning. This analysis is called *GLM analysis* and is described in Section “Assessing Dynamic, Static, and Exponential Contributions (*GLM Analysis*).” We then addressed the adaptation of sensory processing (of Bayesian learning in particular) under predictability in a second analysis (*Predictability analysis*) described in Section “Automatic Context Adaptation of Sound Processing (*Predictability Analysis*).” In this case, we first inverted data in context UC (two runs) to derive estimates of model parameters and these were next used as priors for model inversion in context PC (two runs). In this way, the ON family for one regressor gathers models where its related coefficient could depart from UC prior. The resulting posterior probability map (step 4) thus smartly indicates where and when the cognitive account of sensory processing associated to the regressor is shaped by predictability.

### Statistical Model

We considered a general linear model (GLM) of the form:


(1) $$\begin{array}{c} y=h_{0}X_{0}+h_{dyn}^{BS}X_{dyn}^{BS}+\sum_{i\in \left\{std,dev\right\}}h_{static}^{i}X_{static}^{i}\\ \;+\sum_{i\in \left\{rnk,cs\right\}}h_{exp}^{i}X_{exp}^{i}+\varepsilon \end{array}$$

Where *y* is the trial-by-trial time series measured at a given ECoG location and a particular peristimulus time sample across all trials (for each subject, for each sensor, for each run and for each of the *N_s_* = 120 samples spanning the [–100 +500] ms epoch, *y* is a vector of size *N_t_* = 672 trials). All parameters of the linear combination (denoted ${\text{h}}_{*}^{*}$) are defined as Gaussian random variables and ε is a Gaussian measurement noise. Regressors (${\text{X}}_{*}^{*}$) all consist in trial-wise trajectories, each representing a candidate explanatory factor (Figure 1B). First term in equation (Eq. 1) corresponds to the mean factor, with *X*_0_ being a unit vector. Below, we present the five regressors that we aimed to assess, and that we grouped in three categories:

• *Dynamic regressor* ($X_{\text{dyn}}^{BS}$)

This category involves a learning regressor deriving from an internal generative model that assumes that the brain learns from each stimulus presentation the probability μ to have a deviant to predict the next stimulus category *U* (with *U_k_* = 1 in the case of trial *k* corresponding to a deviant and *U*_*k*_ = 0 in the case of a standard). We define *U*∼*Bern* (μ) with *Bern* the Bernoulli distribution, and μ∼*Beta* (α, β) with α and β the parameters of the *Beta* distribution corresponding to deviant and standard counts at trial *k*, respectively (Eq. 3). Regressor reflects a precision-weighted prediction error at every sound of the oddball sequence, which expresses as a Bayesian Surprise (BS; Ostwald et al., 2012). In short, BS quantifies the belief updating on μ as it corresponds to the Kullback-Leibler divergence between the prior and the posterior ℬ*eta* distributions over μ. At trial *k*, following the observation of sound input *U*_*k*_, it writes:


(2) $$\begin{array}{c} BS\left(U_{k}\right)=\log\left(\frac{\Gamma \left(\alpha_{k-1}+\beta_{k-1}\right)}{\Gamma \left(\alpha_{k}+\beta_{k}\right)}\right)+\log\left(\frac{\Gamma \left(\alpha_{k}\right)}{\Gamma \left(\alpha_{k-1}\right)}\right)\\ \;+\log\left(\frac{\Gamma \left(\beta_{k}\right)}{\Gamma \left(\beta_{k-1}\right)}\right)+\left(\alpha_{k-1}-\alpha_{k}\right)[\psi \left(\alpha_{k-1}\right)\\ \;-\psi \left(\alpha_{k-1}+\beta_{k-1}\right)]+(\beta_{k-1}-\beta_{k})[\psi \left(\beta_{k-1}\right)\\ \;-\psi \left(\alpha_{k-1}+\beta_{k-1}\right)] \end{array}$$

Where Γ and ψ are the Gamma and Digamma Euler functions, respectively. Internal states α, β are updated as well as $X_{\text{dyn}}^{BS}$ is augmented as follows:


(3) $$\left\{ \begin{array}{c} \alpha_{k+1}=U_{k}+e^{-\frac{1}{\tau }}\alpha_{k}\\ \beta_{k+1}=\left(1-U_{k}\right)+e^{-\frac{1}{\tau }}\beta_{k}\\ X_{dyn,k+1}=BS\left(U_{k,}\alpha_{k},\beta_{k},\tau \right) \end{array}\right.$$

Full description of the model is provided in our previous EEG-MEG work (Lecaignard et al., 2021b). As can be seen from equation (Eq. 3) and in Figure 1B (lower panel), standard and deviant counts vary with model parameter τ, a time constant that enables controlling the relative influence of past events in belief updating. It can be viewed as the size of the temporal integration window (or memory span). In our previous EEG-MEG study, a more predictable sequence was found to yield an increase in τ, which is consistent with the idea that the more regular or structured the sensory environment, the more one should rely on past events to form predictions. In the following, since regressor $X_{\text{dyn}}^{BS}$ is the only one to be both dynamic and the output of a generative learning model, it will be called the *dynamic* or the *learning* regressor equivalently.

• *Static regressors* ($X_{\text{static}}^{std},X_{\text{static}}^{dev}$)

We here include two regressors to classify trials according to the actual sensory input (a standard or a deviant sound). $X_{\text{static}}^{std}$equals 1 at every occurrence of a standard stimulus, and 0 at every occurrence of a deviant stimulus. $X_{\text{static}}^{dev}$ is the complementary of $X_{\text{static}}^{std}$ (it is equal to 1-$X_{\text{static}}^{std}$). Although their respective trajectory is not constant (Figure 1B, upper panel), we consider these two regressors as static in the sense that they do not incorporate any time dependency but simply capture stimulus category. They are similar to the ‘change detection’ regressors defined in previous MMN modeling studies (Lieder et al., 2013; Stefanics et al., 2018; Lecaignard et al., 2021b). They indeed get close to the actual definition of the MMN and the way (averaged) oddball responses are traditionally computed, although typical studies usually discard the first standard following a deviant or even sometimes all standards but the one just preceding a deviant, precisely to get rid of time-dependent (dynamic) effects.

• *Exponential regressors* ($X_{\text{exp}}^{rank},X_{\text{exp}}^{cs}$)

Introducing this additional category was motivated by well-established MMN findings, namely that standard responses decrease over stimulus repetitions (Grill-Spector et al., 2006) and that the MMN amplitude increases as the number of standards preceding a deviant (chunk size) increases (Sams et al., 1983). Note that these inter-trial modulations cannot be predicted by the above static regressors ($X_{\text{static}}^{std},X_{\text{static}}^{dev}$), but could coincide with the predictions from the above dynamic (learning) factor, as was found in Lecaignard et al. (2021b). However, for a fair examination of brain signal dynamics in relation to these MMN findings, we considered two additional regressors accounting for standard repetition effects and deviant history, while not reflecting some output from a specific cognitive process. They concern the rank of stimulus repetition, where at trial *k*, *rank*(*U*_*k*_) is defined as the within-chunk number of presentation of current stimulus *U_k_*, and chunk size, where *cs*(*U*_*k*_) is the size of the current chunk. Both rank and chunk size can take *n* values in the 2–8 range (they are defined ad-hoc as no generative model is involved here). We used exponential rather than linear factors because of recent EEG findings in the visual modality that showed that these regressors clearly best explain the repetition-suppression effect and its modulation by the number of standard repetitions (Stefanics et al., 2020). Thus, we defined regressors $X_{\text{exp}}^{rank}$ and $X_{\text{exp}}^{cs}$ as the normalized mean-centered exponential function of trial rank and trial chunk size, respectively. At trial *k*, we have:


(4) $$\left\{ \begin{array}{c} X_{exp}^{rank}\left(U_{k}\right)=\frac{\exp\left(rank\left(U_{k}\right)\right)-1/n\sum_{i=1}^{n}\text{exp}\left(rank\left(i\right)\right)}{\text{exp}\left(\text{max}\left(rank\left(U\right)\right)\right)}\\ X_{exp}^{cs}\left(U_{k}\right)=\frac{\exp\left(cs\left(U_{k}\right)\right)-1/n\sum_{i=1}^{n}\text{exp}\left(cs\left(i\right)\right)}{\text{exp}\left(\text{max}\left(cs\left(U\right)\right)\right)} \end{array}\right.$$

It should be noticed that since deviant is of rank 1, the only way to account for different brain responses to deviant and standard following a deviant is to involve a mixture of the rank regressor with either the chunk size or the static regressors (our modeling procedure is precisely equipped to test such hypothesis). As can be seen in Figure 1B, the rank regressor (middle panel, pink trace) shows a possible dynamics for the expected standard-to-standard variations (as amplitude increases over repetitions, we would expect a negative posterior estimate for coefficient $h_{\text{exp}}^{rank}$) that differs from the BS one (lower panel). Similarly, chunk size regressor (purple) assigns different amplitude to deviants depending on local past experience. The two exponential factors thus enable testing a conservative approach as the learning factor will now be proved explanatory only if it captures trial-wise fluctuations that are not captured by these more traditional factors (May and Tiitinen, 2010).

In sum, our model (that we denote as the full model) enables mixing competing trial-based covariates to refine the spatio-temporal description of cognitive processes engaged during the current oddball sequence exposure. The static category contrasts with the other two as related regressors $X_{\text{static}}^{std}$ and $X_{\text{static}}^{dev}$ are not equipped to capture time-dependent or trial order effects. Besides, the dynamic and exponential categories differ in their predictions of inter-trial fluctuations: dynamic regressor $X_{\text{dyn}}^{BS}$ is computed as the output of a generative model implementing the learning of stimulus regularities whereas the exponential regressors are not directly computationally interpretable ($X_{\text{exp}}^{rank}$ and $X_{\text{exp}}^{cs}$ do not map onto cognitive mechanism). In the following, we provide in detail the modeling approach that we used to assess the contribution of each regressor over space and time to the ECoG data.

### Assessing Dynamic, Static, and Exponential Contributions (*GLM Analysis*)

This first analysis aims at characterizing sensory processing during an oddball sequence (whatever the predictability manipulation, considering both contexts UC and PC), and testing in particular the Bayesian learning of deviant probability that we could evidence previously using EEG and MEG (Lecaignard et al., 2021b). We evaluate the relevance of each linear regressor (n = 6; $X_{\text{dyn}}^{BS}$, $X_{\text{static}}^{std}$, $X_{\text{static}}^{dev},$$X_{\text{exp}}^{rank},$$X_{\text{exp}}^{cs}$ and *X*_0_ = 1) to account for trial-to-trial fluctuations. Each evaluation involves nested versions of the full model, that we compare using Bayesian model comparison and family-level inference (Penny et al., 2010).

First, we here describe the model space for this *GLM analysis*, followed by a description of model inversion. Next, we present the family-level inference procedure performed for each regressor to assess its contribution to the observed data. Finally, we provide the details of two additional studies that were conducted to refine our analysis. The first one is based on simulated data and aims at controlling the ability of our approach to separate models (to infer the *true* generative model). The second consists in replicating the GLM analysis without including the learning regressor to test the specificity of its trial-by-trial dynamics compared to those of exponential regressors.

#### Model Space

Recently, a novel approach proved efficient to test the relevance of GLM factors, that frames this question in terms of model comparison (Friston et al., 2016, 2018). Precisely, for each regressor, we consider two GLM: one where the regressor is present (or switched ON) and one where it is absent (or switched OFF), using non-null and null coefficient $h_{*}^{*}$ in equation (Eq. 1), respectively. Applying the ON/OFF scheme to all regressors, we could build a model space with all possible combinations (*N*_*m*_ = 2^6^ = 64 models), depicted in Figure 2 (right panel). As we shall see, evaluating the relevance of one regressor amounts to comparing in a Bayesian model comparison fashion the 32 models where it is ON with the 32 other ones where it is OFF (Figure 2, panel “Model Comparison”).

#### Model Inversion

First, we fitted the full model to the ECoG signals in both contexts PC and UC, for each retained data point (that is, for each of the *N_s_* = 120 peri-stimulus time sample of each accepted sensor). We used a Variational Bayes (VB) scheme implemented in the VBA toolbox (Daunizeau et al., 2014). Gaussian prior distributions were employed for every coefficient parameter, all with zero mean and non-null variance ($h_{*}^{*}$∼𝒩(0,5)). We use a similar Gaussian prior for the learning parameter (*log*(τ)∼𝒩(2,5)). Data involved UC and PC runs (two runs per condition) and inversion was achieved in the following fashion: each run (*N_t_* = 672 trials) was treated independently (model fit always starts with the above-mentioned priors) but posterior estimate of each regressor coefficient accounts for the entire set (the four runs). Bad trials were ignored to avoid contaminating model fit with noisy signals but corresponding stimuli still entered model dynamics as they were observed by the brain. At convergence of the VB scheme, model inversion provides the Free Energy approximation of the log-model evidence (Friston et al., 2007), the percentage of explained variance afforded by the model (denoted *R2*) and the posterior distributions of model parameters ($\tau ,h_{0},h_{\text{dyn}}^{BS}$, $h_{\text{static}}^{std}$, $h_{\text{static}}^{dev}$, $h_{\text{exp}}^{rank}$, $h_{\text{exp}}^{cs}$). Data point selection was based on full model responsiveness, defined using a threshold on R2 (5%; more details about data selection is provided in Section “Data Point Selection”). Next, regarding the 63 nested models, we employed Bayesian Model Reduction (BMR; Friston et al., 2016) to derive analytically the (reduced) free energy and posterior estimates for each model from those obtained with the full model inversion. BMR affords a great gain in terms of computational resource (only the full model is to be inverted) and has been shown to provide better results than VB nested model inversions that involve iterative optimization procedure, with possibly the undesirable issue of local minima convergence (Friston et al., 2018). In practice, for each switched-OFF regressor, prior distribution of the corresponding regression coefficient was set to $h_{*}^{*}$∼𝒩(0,0) with the null variance forcing posterior estimate to stick to the null prior mean. Prior distribution for the switched-ON regressors was equal to ($h_{*}^{*}$∼𝒩(0,5)).

#### Family-Level Inference

For each regressor, model comparison relied on family-level inference to compare the ON and OFF families of models, defined by grouping the 32 ON models and the 32 OFF models, respectively (Figure 2, panel “Model comparison”). Family comparison was based on the *N*_*m*_ = 2^6^ free energies described above (full and reduced values). Applying the softmax function to these free energies enables computing the posterior probability of the ON family. The larger the ON posterior probability, the more likely the corresponding regressor contributes to the observed data. We performed ON/OFF family comparison for each regressor to derive the 6 ON posterior probabilities. They enabled us to examine the relevance of each corresponding hypothesis for sensory processing. Importantly, this scheme (6 ON/OFF family comparisons) was performed independently at every responsive peri-stimulus time point at every good electrode, in the aim to finely describe spatio-temporally oddball processing on a single-trial basis. For sake of clarity, the notation “family $X_{*}^{*}$ = ON” will be used in what follows to differentiate between families when necessary.

Finally, Bayesian model averaging (BMA; Penny et al., 2006) provides the posterior estimates of model parameters averaged across model space (with model-evidence weighting based on the full and reduced free energies).

#### Model Separability (Simulation Study)

We investigated the ability of the above-mentioned procedure to recognize the respective contribution of each regressor, in particular with the present case of single-trial signals (as will be seen, the full model inversion yields rather low goodness-of-fit). To do so, we considered BMA posterior estimates of regressor coefficients measured at a particular time point on one electrode in a given participant. The full model was used with these values to generate 100 datasets, each made of two runs per context (UC and PC) using the exact stimulus sequences delivered to that patient. Critically, Gaussian noise was added to the synthetic data and its variance was adjusted so that the percentage of variance explained by the full model when inverting this synthetic set was of the same order of magnitude as the one measured with the real data. Values of R2 (from observed and synthetic data inversion) as well as measurement noise precision are provided in Table 2. We refer to these simulated data as the full data with regard to their generative model. We then generated another 100 datasets using only a dynamic contribution ($\tau ,h_{0,}h_{\text{dyn}}^{BS}$were equal to the BMA values while $h_{\text{static}}^{std}$, $h_{\text{static}}^{dev},$$h_{\text{exp}}^{rank}$ and $h_{\text{exp}}^{cs}$ were set to 0); we refer to them as the learning data. Last, we generated 100 datasets using only static and exponential contributions ($h_{\text{static}}^{std}$, $h_{\text{static}}^{dev},$$h_{\text{exp}}^{rank}\mathrm{and}h_{\text{exp}}^{cs}$ were equal to the BMA values while $h_{\text{dyn}}^{BS}$was set to 0); we refer to them as the non-learning data. Each of the 300 datasets was confronted to our procedure (full model inversion, BMR and family-level inference). Within each generative model case (full, learning, non-learning), we conducted family model comparison for each regressor. We used a random-effect (RFX) model (Penny et al., 2010) to treat independently each of the 100 simulations.

**TABLE 2:** Parameters and results obtained in the simulation study.

	ECoG inversion			Simulations		Full	Learning	Non-learning
				**Noise precision**		0.004	0.004	0.004
Case 1	**R2**		14.7	**R2 (mean)**		13.0	6.9	2.0
	**Pp** $X_{\text{dyn}}^{BS}$ / $X_{\text{static}}^{std}$		> 0.99 / 0.86	$X_{\text{dyn}}^{BS}$	**Pp range**	0.4–1.0	0.4–1.0	0.0–0.1
	**BMA**	*log*(τ)	3.853		**RFX**	1.0	1.0	0.0
		$h_{\text{dyn}}^{BS}$	–0.128	$X_{\text{static}}^{std}$	**Pp range**	0.3–0.7	0.0–0.0	0.0–1.0
		$h_{\text{static}}^{std}$	0.006		**RFX**	1.0	0.0	1.0

				**Noise precision**		0.003	0.003	0.003
Case 2	**R2**		12.6	**R2 (mean)**		12.2	12.1	0.2
	**Pp** $X_{\text{dyn}}^{BS}$ / $X_{\text{static}}^{std}$		> 0.99 / 0.002	$X_{\text{dyn}}^{BS}$	**Pp range**	0.4–1.0	0.4–1.0	0.0–0.0
	**BMA**	*log*(τ)	2.870		**RFX**	1.0	1.0	0.0
		$h_{\text{dyn}}^{BS}$	–0.094	$X_{\text{static}}^{std}$	**Pp range**	0.0–0.0	0.0–0.0	0.0–0.0
		$h_{\text{static}}^{std}$	0.000		**RFX**	0.0	0.0	0.0

				**Noise precision**		0.002	0.002	0.002
Case 3	**R2**		15.9	**R2 (mean)**		16.1	0.2	16.4
	**Pp** $X_{\text{dyn}}^{BS}$ / $X_{\text{static}}^{std}$		0.15 / > 0.99	$X_{\text{dyn}}^{BS}$	**Pp range**	0.0–0.1	0.0–0.1	0.0–0.1
	**BMA**	*log*(τ)	2.761		**RFX**	0.0	0.0	0.0
		$h_{\text{dyn}}^{BS}$	0.000	$X_{\text{static}}^{std}$	**Pp range**	0.0–1.0	0.0–0.0	0.0–1.0
		$h_{\text{static}}^{std}$	0.026		**RFX**	1.0	0.0	1.0

**FIGURE 3:**
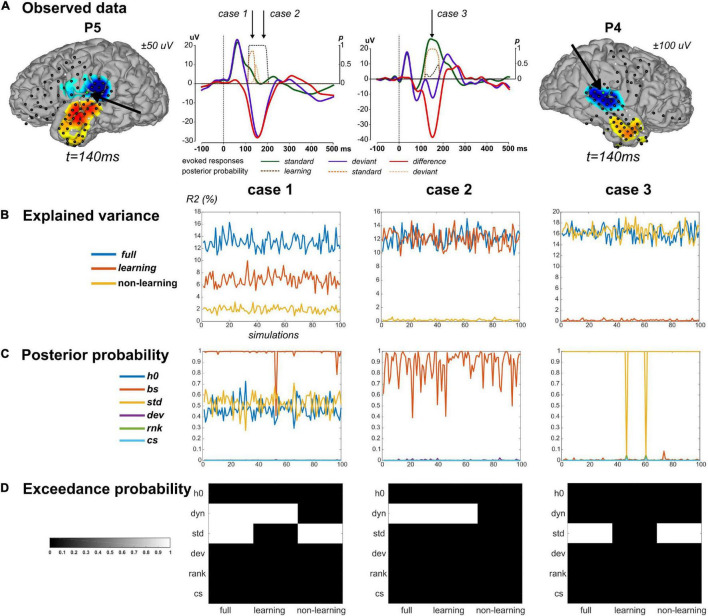
Simulation findings. **(A)** Simulations were based on parameter values inferred from ECoG data fitting. Three cases were considered. Case 1 and Case 2 concern one sensor in P5, highlighted on the cortical surface (same display as in Figure 5A). Corresponding evoked responses at this sensor (average response across contexts UC and PC), for standard (green), deviant (purple) stimuli, and their difference (red), and posterior probabilities at peri-stimulus samples for the learning (brown), standard (orange) and deviant (yellow) regressors suppress (color code provided on the figure). Case 3 derived from one sensor in P4 (right). Cases are presented in columns in panels **(B–D)**. **(B)** Percentage of explained variance when fitting the full model to 100 simulated data (x-axis) generated from the full (blue), learning (red) and non-learning (yellow) models. **(C)** Posterior probability of each regressor (following the legend provided) for each simulated data with the full model. **(D)** Posterior exceedance probability for each regressor (y-axis) computed from family-level inference (RFX) performed over the 100 simulations generated from the full, learning and non-learning models (x-axis).

*Three simulation analyses (Case 1, Case 2 and Case 3) were performed using different model parameter values inferred from ECoG data (see Figure 3A). For each case (rows), specific findings from ECoG inversion are provided (left): explained variance of full model fitting (R2) expressed as percentage, family ON posterior probability (Pp) for the learning and standard regressors, and BMA estimates of model time constant (τ), learning and standard regressor coefficients. Simulation results obtained from fitting the synthetic datasets generated with the full, learning, and non-learning GLM are provided (right). Measurement noise precision corresponds to the inverse variance of the Gaussian noise added to the synthetic data. R2 corresponds to the average over the 100 simulations. Pp range: minimum and maximum posterior probability values of family ON observed over the 100 simulations. RFX: posterior exceedance probability of family ON resulting from model comparison performed over the 100 simulations. $X_{dyn}^{BS}$, $X_{static}^{std}$, $h_{dyn}^{BS}$, $h_{static}^{std}$ and τ correspond to GLM parameters described in the main text.*

We applied this scheme to three data points in particular (Figure 3A) at which we found strong evidence for both standard and learning (case 1, a posterior temporal electrode in P5 at 130 ms), for learning only (case 2, posterior temporal electrode in P5 at 180 ms), and for standard only (case 3, posterior temporal electrode in P4 at 150 ms). Values of R2 and BMA estimates of $\tau ,h_{\text{dyn}}^{BS}$ and $h_{\text{static}}^{std}$ obtained from real data fitting are provided in Table 2. Applying the above-described modeling procedure to the resulting synthetic datasets, we would conclude in favor of model separability afforded by our modeling approach if for each case we could select the true regressor(s) and reject the null ones in the full, learning and non-learning RFX analyses.

The selected values for noise precision yielded R2 values that were found on average over simulations close to the observed data value, suggesting that we succeeded in generating similar conditions of data fitting between predicted and observed conditions (Table 2). For each case, RFX family inference (Figure 3D) indicated that contributions from dynamic and standard regressors could be retrieved when present in the true model (posterior exceedance probability = 1.0). In all three cases however, the different posterior probabilities for family $X_{\text{dyn}}^{BS}=ON$ obtained over simulations showed values between 0.4 and 1.0 (Figure 3C and Table 2). It is important to acknowledge this variability and keep in mind that real data inversion could well yield a posterior probability value within that range. Regarding the learning data, very poor goodness-of-fit was found over simulations (mean R2 = 0.2%) in case 3. This was expected as these data were generated with no contribution from the dynamic regressor. We obtained similar results with the non-learning data in case 2 (mean R2 = 0.2%). Importantly, as can be seen in Figure 3, learning and non-learning data inversions yielded RFX family comparison that always indicated strong evidence for the true contributing regressor and poor evidence for the non-contributing ones. Overall, these findings demonstrate the reliability of this scheme (full model inversion, BMR and family-level inference) for single trial data analysis.

#### Specificity of the Bayesian Surprise Dynamics

The learning regressor was found necessary to account for trial-by-trial data in a spatially restricted but robust fashion (posterior probability of ON family ≥ 0.9), while the exponential ones could be clearly rejected. To better understand this effect, we next examined the specificity of the BS time course over trials in comparison to the exponential ones (Figure 1B). In other words, we assessed whether the exponential contributions could provide a better fit when taking the learning factor out of the model (in this way, we derive a model space comparable to the one used in the study by Stefanics et al. (2020), where the exponential model was found winning). We thus ran another time the ON/OFF family comparison (Figure 2, step 3) for each regressor and at each responsive data point, over the subset of model space where $X_{\text{dyn}}^{BS}$was absent (*N_m_* = 32). Increased evidence for the $X_{\text{exp}}^{rank}=ON$ and $X_{\text{exp}}^{cs}=ON$ families would indicate the relevance of a dynamic trajectory, be it exponential or learning-based. On the contrary, similar rejection of exponential regressors as when learning is present would point to the BS specificity and strengthen the finding of a contribution of the learning regressor.

### Automatic Context Adaptation of Sound Processing (Predictability Analysis)

This second analysis pertains to the modulatory effect of predictability on learning, an effect measured at the group-level using EEG-MEG recordings. In this previous work, we considered a learning model that expresses as the present GLM (Eq. 1) reduced to the *X*_0_ and the $X_{\text{dyn}}^{BS}$ contributions. Each context (UC, PC) was treated separately and we tested for a difference in the resulting posterior estimates using an ANOVA. Such a predictability effect could be observed at the group-level as a difference in the posterior estimates of the learning parameter τ between contexts (we found τ_*PC*_ > τ_*UC*_).

Here, to assess the difference in auditory processing between the two contexts, we adopt a different procedure inspired from typical analysis using dynamic causal models, where one experimental condition is defined as the basic process performed by the brain while the other condition is treated as perturbing this basic state (Garrido et al., 2009a; Kiebel et al., 2009). The strength of such approach lies in the fact the identification of specific model parameter(s) that capture(s) the difference between conditions is itself informative about the mechanisms behind such different processing. Not only this approach accounts very well for the expected predictability effect that we seek (an automatic adaptation of typical oddball processing through the modulation of the learning process) but also, from a methodological perspective, testing it can be handled very nicely with the ON/OFF family-level inference procedure deployed in the GLM analysis.

Precisely, we here started fitting only the UC data using the same priors as defined in the previous section, and the resulting estimates of model parameters (regressor coefficients and learning parameter τ) enabled to characterize a baseline for oddball processing. These values were next used as priors to fit the GLM with the PC data; here again the full model was inverted using a variational approach and the nested ON/OFF models were treated using BMR. The ON/OFF family comparison scheme was applied to model parameters (this time including τ) in context PC. Importantly, since priors were no longer null (depending on UC data), the ON/OFF family comparison now enables testing the conformity/departure from priors resulting from the posterior estimates after fitting UC data, which speaks to the absence/presence of predictability effect. In sum, we here assess whether model parameters (the regressor coefficients, and the learning rate τ) in context PC should depart from baseline (UC) value in order to account for learning in a predictable context.

In more detail, we restricted the analysis to data points where evidence for learning was supported in the previous analysis. We chose a threshold of 0.75 on family $X_{\text{dyn}}^{BS}=ON$ posterior probability to that aim. For a fair examination of all predictability effects, we also included data points showing evidence for other contributing regressors (using the same threshold on posterior probability). In sum, all data points showing at least one regressor (except mean regressor *X*_0_) for which posterior probability of the ON family was larger than threshold was included in the present analysis.

Starting with context UC, we applied the procedure described in Figure 2 (step 1 to step 3) to derive BMA posterior estimates for every regressor coefficient. To obtain a fine estimate of the learning parameter in that context, we used those posterior estimates as priors over corresponding parameters in a dedicated inference where (h_*_∼𝒩(μ_h*,*BMA*_,5)) while keeping an uninformative log-normal prior over the learning parameter itself (*log*(τ)∼𝒩(2,5)). The resulting posterior mean estimates were then used as prior means for subsequent inversions in context PC. Regarding prior variance, an important distinction was made between the learning and static investigations. For the former, we expected predictability to affect learning parameter but not the regressor coefficient. This is because τ is an evolution parameter involved in the learning process (it shapes the effect of learning over trials) while $h_{\text{dyn}}^{BS}$ is an observation parameter used to map hidden activity (here the BS) onto actual measurements (at the sensor level). Contrary to the evolution parameter, this observation one is meant to capture biophysical properties of the data generative process that are unrelated to the cognitive processes at play. This led us to set the following prior variance (for convenience, notation $h_{\text{dyn}}^{BS}$ has been reduced to *dyn* in subscripts):


(5) $$\begin{array}{c} \sigma_{\tau }=\left\{ \begin{array}{cc} 5\;ifX_{dyn}^{BS}=ON\; & \\ 0\;otherwise\; & \end{array}\right.\\ \sigma_{dyn}=0 \end{array}$$

For regressors $X_{\text{static}}^{std}$, $X_{\text{static}}^{dev},$$X_{\text{exp}}^{rank}\mathrm{and}X_{\text{exp}}^{cs}$, which do not include any evolution parameter but a single observation one ($h_{*}^{*}$), prior variances were set as follows:


$$\sigma *=\left\{ \begin{array}{cc} 5\; & ifX_{*}^{*}=ON\\ 0\; & otherwise \end{array}\right.$$

Similarly, we did not allow for offset parameter *h*_0_ to vary between contexts, considering the prior distribution h0∼𝒩(μ,0,BMA0).

Following full model inversion in PC using these adjusted prior distribution, the BMR and family-level inference procedure (Figure 2, steps 2, 3) was performed to assess the relevance of the evolution (τ) and observation ($h_{*}^{*}$) parameters. This procedure was run separately for each parameter category. For the evolution parameter τ, at every data point that showed a significant learning effect in the previous GLM analysis ($p\left(X_{\text{dyn}}^{BS}=ON\right)\geq 0.75$), family-level inference was run over a model space with 2 models (τ being ON/OFF). Regarding the observation parameters, similarly we selected data points that showed at least a significant contribution of the static or the exponential models in the previous GLM analysis. As the number of these effects varies from one data point to another, the model space was therefore specific to each of them (it comprises 2^*n*^ models with *n* the number of free parameters or, equivalently, the number of significant effects at that particular data point).

We also considered testing the predictability effect on the MMN component, as a significant reduction under context PC was measured at the group-level using each EEG and MEG modality separately (Lecaignard et al., 2015, 2021b). To that aim, at the individual level, we focused on post-stimulus time points in 100 and 200 ms where the MMN could be identified in all participants (Figure 5B). For each sensor that exhibited a learning effect in the *GLM analysis* (posterior probability larger than 0.75) at least in one of these time points, we averaged the [100 200] ms data for each accepted single trials. The resulting values were then examined using an unbalanced two-way ANOVA with a factor of stimulus type (standard, deviant) and a factor of context (UC, PC) in MATLAB (R2017b, The MathWorks Inc.).

### Data Point Selection

**FIGURE 4:**
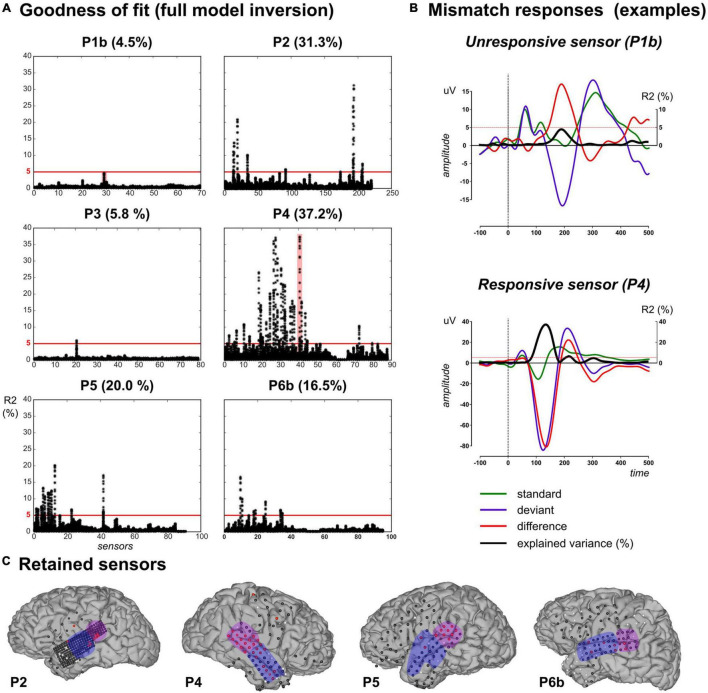
Data selection based on goodness-of-fit in the full model inversion. **(A)** For each patient, explained variance (R2 percentage value) measured at each sensor (*x*-axis) for each peri-stimulus sample inversion (N_s_ = 120 black dots covering the [–100, 500] ms epoch per sensor). **(B)** Evoked responses elicited by standard (green), deviant (purple) and their difference (red) for two unresponsive (in P1b, upper plot) and responsive (in P4, lower plot) sensors. These two sensors are highlighted (red shaded areas) in panel **(A)**. Black trace indicates the R2 time-course. Red horizontal lines indicate the 5% threshold. **(C)** Cortical surface with sensor overlay in the four patients included in the present work. Selected sensors based on R2 thresholding are depicted in red, unresponsive and bad (rejected) sensors are in black and white, respectively. The present findings were measured in the anterior (blue) and posterior (purple) temporal regions.

Electrocorticographic grids provide a large number of electrodes, in particular high-density grids such as the one used with P2. For the sake of tractable computations as well as not to draw conclusions out of very poor model fits, we restricted the above analysis to ECoG electrodes with a fair amount of explained variance. Indeed, single-trial modeling approaches are quite recent, with no established standard regarding the expected explained variance contrary to more conventional ERP analysis. In a recent ECoG study (Sedley et al., 2016), a linear model composed of learning-based regressors (surprise, prediction error and precision trajectories) was applied to time-frequency data and resulted in an explained variance of the order of 2% or less, across subjects and electrodes. Here we selected data points based on the percentage of explained variance when fitting the full model to the pooled UC and PC data. Inspection of R2 values obtained at each sample, each sensor (Figure 4A) shows that R2 reached values up to 37.2%.

We further computed the individual evoked responses to standard and deviant sounds, and their difference exhibiting the MMN. Overlaying R2 time series on these responses (resulting from model inversions at each peri-stimulus time sample) revealed that maximum R2 values did coincide with electrodes and latencies showing the MMN (Figure 4B). Based on this qualitative investigation, we decided to apply an R2 threshold of 5% for data selection. This value resulted in the rejection of all data from P1a and P1b. In patient P3, only one electrode proved above-threshold (5.8%; over 4 consecutive time samples from 180 to 195 ms, see Supplementary Material). Finally, 10, 28, 11, 18, and 7 sensors fulfilled the selection criterion for patients P2, P4, P5, P6a, and P6b, respectively.

The above-described simulation study could confirm the validity of this data selection procedure with a 5% threshold for explained variance to separate models reliably.

All selected electrodes were found distributed over temporal regions (except for one parietal sensor in P4). In the following, for convenience, we present the results on those electrodes, which we split into two groups: the anterior and the posterior part of the temporal lobe, respectively (Figure 4C).

## Results

**FIGURE 5:**
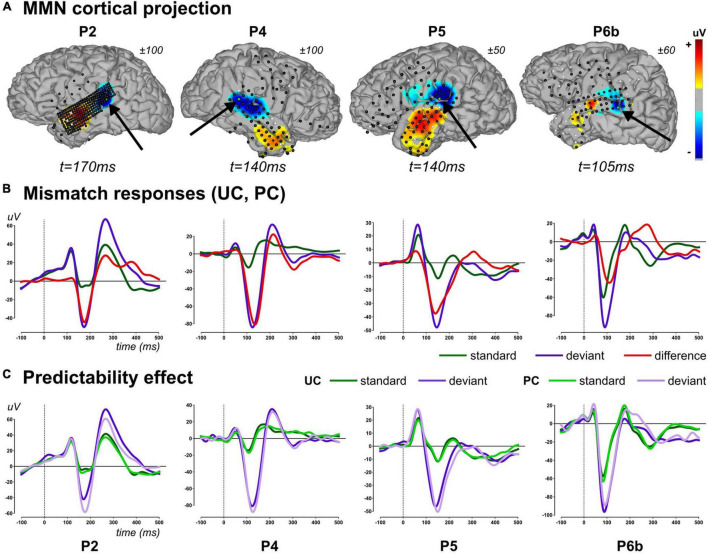
Mismatch evoked responses (2–20Hz). **(A)** Projection of difference responses (deviant–standard) around the MMN peak onto cortical surface (linear projection based on sensor-to-mesh distance), for each patient (columns). Latency and amplitude range are provided for each patient. Sensor overlay: black and white dots represent good and bad sensors, respectively. For each cortical map, black arrow points to a relevant electrode (green dot) showing an MMN, whose evoked activity is provided in lower panel. **(B)** Average evoked activity across contexts (UC and PC) for standard (all of them including those of rank 1), deviant stimuli, and their difference for each patient (column), at a particular electrode (highlighted in panel **A**). **(C)** Evoked standard and deviant responses at the same electrode in context UC and PC. Panels **(B,C)**: traces are baseline corrected (–100 to 0 ms) and follow the color code provided.

We report findings measured in four patients (P2, P4, P5, and P6b), first identifying the relevant explanatory variables and their spatio-temporal mapping (*GLM analysis*), and then testing for the effect of our experimental manipulation (*Predictability analysis*). Typical evoked responses to standard (occurring at any position within a chunk) and deviant stimuli as well as their differences are shown in Figure 5B, along with standard and deviant waveforms measured in each context separately (UC, PC) in Figure 5C.

Results in both sections below were obtained from selected data points (see “Materials and Methods” section). We do not report findings regarding the constant regressor (coefficient *h*_0_in Eq. 1); they are provided as Supplementary Material (Supplementary Figure S4).

### GLM Analysis

Responsive data points (R2 ≥ 5%) were all found in the post-stimulus interval, at samples exhibiting the MMN in the following time windows: 145–325 ms in P2, 100–335 ms in P4 (one anterior temporal sensor showed also later responsiveness in 405–470 ms), 115–290 ms in P5, and 90-215 ms in P6b. We start by presenting the family-level inference results obtained with the ECoG data (UC and PC contexts) in the aim to assess the presence of each regressor in the GLM. We next show the effect of switching off the contribution of the dynamic regressor onto the estimated contribution of the static and exponential covariates in order to test if the latter could compensate for the BS absence due to the alternative dynamics they entail (Figure 1B).

#### GLM Analysis (*Fitting UC and PC Data*)

**FIGURE 6:**
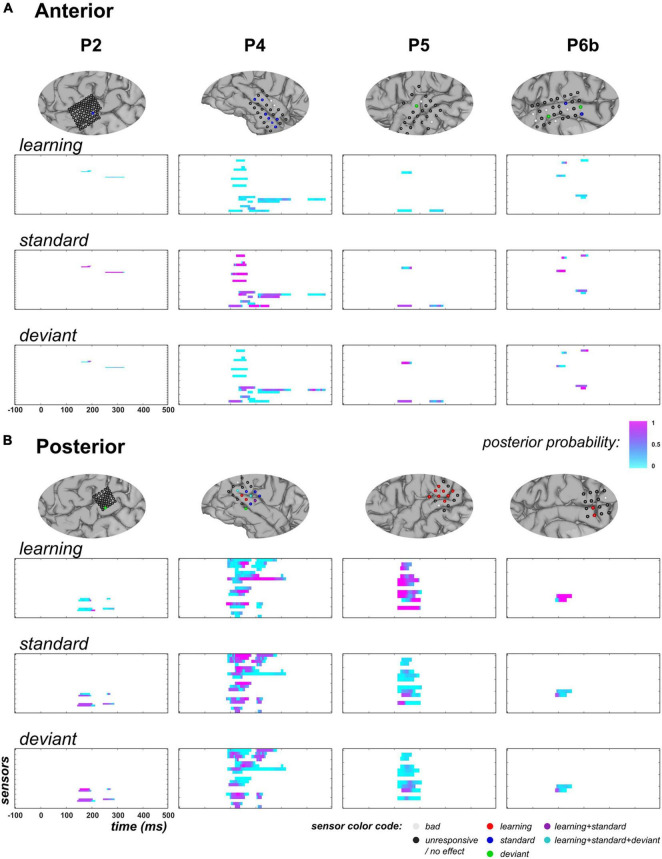
GLM findings at temporal electrodes (across contexts, UC and PC). **(A)** Anterior overlay. Top row: for each subject (columns), zoomed view of cortical mesh with anterior temporal electrodes (following the clustering depicted in blue in Figure 4C). Electrodes exhibiting a posterior probability larger than 0.9 in the 50–250 ms time window (at least one sample) for one or multiple regressors are colored following the code provided at the bottom right of the figure. Rows 2 to 4: family-level inference for regressor ${\text{X}}_{\text{dyn}}^{\text{BS}}$, ${\text{X}}_{\text{static}}^{\text{std}}$, and ${\text{X}}_{\text{static}}^{\text{dev}},$ respectively. Each graph represents the posterior probability of family $X_{*}^{*}=\text{ON}$, measured at each peri-stimulus sample. **(B)** Posterior overlay. Same display, with electrodes in posterior clusters (purple cluster in Figure 4C).

Figure 6 shows the posterior probability of families $X_{\text{dyn}}^{BS}=ON$, $X_{\text{static}}^{std}=ON$, and $X_{\text{static}}^{dev}=ON$ measured at responsive time points in the anterior and posterior temporal clusters. In the anterior region, there were 4, 11, 2, and 5 responsive sensors (showing at least one sample with full model inversion R2 ≥ 5%) in P2, P4, P5, and P6b, respectively. Across the four subjects, the learning regressor ($X_{\text{dyn}}^{BS}$) was not found relevant at most responsive data points (median value of posterior probability: 0.09), with only three data points showing posterior probability larger than 0.5 (one sensor in P2, from 230 to 235 ms, *p* > 0.78; one sensor in P6b at 130 ms, *p* = 0.81). In contrast, strong evidence for the standard regressor ($X_{\text{static}}^{std})$ was measured predominantly (median value = 0.72). Posterior probability was found larger than 0.9 over at least one time point in 2/4, 7/11, and 2/5 sensors in P2, P4, and P6b, respectively (depicted in blue in Figure 6A). Regarding the deviant regressor ($X_{\text{static}}^{dev})$, posterior probability median was found equal to 0.26; data points showing values exceeding 0.9 could be found in P4 (one sensor at 425 ms), P5 (one sensor from 135 to 150 ms) and P6b (2 electrodes, from 210 to 215 ms, and from 190 to 205 ms, respectively) (depicted in green in Figure 6A). Concerning the exponential covariates ($X_{\text{exp}}^{rank},X_{\text{exp}}^{cs})$, they both showed low posterior probabilities across patients. Regarding $X_{\text{exp}}^{rank}$, maximum posterior probability did not exceed 0.29 in all patients, but P6b (0.87 at 170 ms). Similarly, maximum posterior probability of $X_{\text{exp}}^{cs}$ was smaller than 0.39 in all patients, but P2 (0.85 at one sensor, from 265 to 270 ms).

In the posterior temporal clusters, we report 4, 15, 9, and 2 responsive electrodes in P2, P4, P5, and P6b, respectively. No clear evidence supporting family $X_{\text{dyn}}^{BS}=ON$ was found in P2 (maximum value of 0.83, at one sensor from 200 to 215 ms) but in P4, P5, and P6b (each with maximum value of 1.0; 5/15, 8/9, and 2/2 electrodes above 0.9, respectively; depicted in red in Figure 6B). Across subjects, this learning effect was spanning from 85 to 215 ms (one sensor in P4 also showed posterior probability larger than 0.9 from 140 to 270 ms). For the static category, P4 showed 7/15 sensors with posterior probability larger than 0.9 over at least one time point but this effect was not found in the other three patients: maximum probability for $X_{\text{static}}^{std}=ON$ was equal to 0.72, 0.86, and 0.72 in P2, P5 and P6b, respectively. Regarding the deviant regressor, its contribution was found relevant in 1/4 sensor in P2 (*p* > 0.92 from 155 to 185 ms) and 3/15 sensors in P4 (*p* > 0.91 from 125 to 135 ms; *p* = 0.95 at 130 ms; *p* > 0.98 from 150 to 155 ms) but not in P5 and P6b (maximum posterior probability of 0.72 in both cases). In patient P4, 3 electrodes revealed learning and static effects (depicted in purple and cyan in Figure 6B) but not occurring at the same latency. For the exponential models, two patients disclosed an effect for $X_{\text{exp}}^{rank}=ON$: in P2, posterior probability was larger than 0.94 in 3/4 sensors from 165 to 185 ms, and in P5, it was larger than 0.91 in 1/9 sensor from 150 to 170 ms. No evidence could be suggested in P4 and P6b as maximum values were equal to 0.19 and 0.02, respectively. For family $X_{\text{exp}}^{cs}=ON$, posterior probabilities were all measured below 0.64, 0.06, 0.37 and 0.01 in P2, P4, P5 and P6b, respectively.

#### Specificity of Bayesian Surprise Dynamics

As the above findings supported the learning of the deviant probability (hence a dynamic process) in the posterior temporal region, we next examined if the exponential regressors ($X_{\text{exp}}^{rank}$ and $X_{\text{exp}}^{cs}$) would be sufficient to capture this dynamics or whether the proposed learning dynamics would still be required to better explain the data.

**FIGURE 7:**
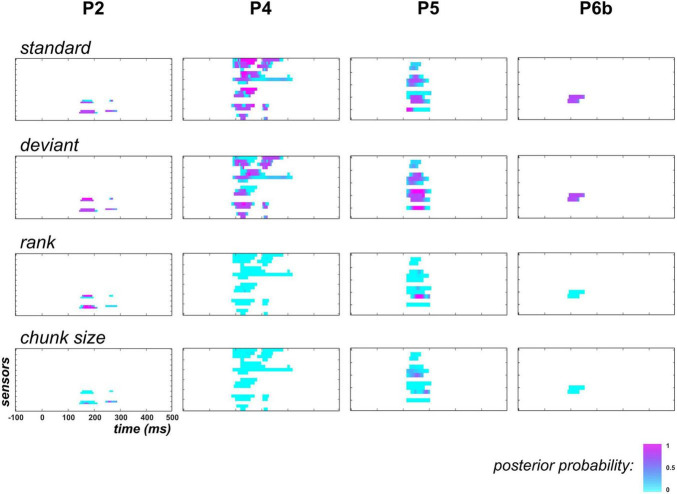
Family-level inference in posterior temporal region when learning is removed from the GLM (UC and PC). Following the display in Figure 6, each individual map shows the posterior probability at posterior temporal sensors and at each peri-stimulus time point for families ${\text{X}}_{\text{static}}^{\text{std}}=\text{ON}$ (**top row**), ${\text{X}}_{\text{static}}^{\text{dev}}=\text{ON}$ (upper central), ${\text{X}}_{\text{exp}}^{\text{rank}}=\text{ON}$ (**lower central**) and ${\text{X}}_{\text{exp}}^{\text{cs}}=\text{ON}$ (**bottom**).

Results are shown in Figure 7. First, it should be noticed that in P2, family-level inference for each regressor (except $X_{\text{dyn}}^{BS}$) revealed similar results as the full model space analysis (this can be seen for $X_{\text{static}}^{std}$ and $X_{\text{static}}^{dev}$ by comparing posterior probability maps between Figures 6, 7). This is because data in this patient did not support the learning model (poor evidence for $X_{\text{dyn}}^{BS}=ON$). Based on findings in P4, P5 and P6b where it was found relevant, we see that the exponential hypothesis could be rejected: across these patients, median posterior probability was equal to 0.005 and 0.004 for families $X_{\text{exp}}^{rank}=ON$, and $X_{\text{exp}}^{cs}=ON$, respectively. On the contrary, the absence of learning contribution tend to increase the estimated contribution of the static family, as median posterior probability increases from 0.26 to 0.61, and from 0.14 to 0.55 for the standard and deviant regressor, respectively (over P4, P5, and P6b). However, this increase remains limited as when focusing on data points with strong learning evidence ($p\left(X_{\text{dyn}}^{BS}=ON\right)\geq 0.9$), these median values were found to change from 0.07 to 0.33 and from 0.03 to 0.40 for the standard and the deviant regressor, respectively.

### Predictability Effect

In this second analysis, we test the hypothesis of an automatic adaptation of sound processing in the predictable context. We first report the analysis of the MMN component, followed by a presentation of the single-trial modeling findings.

In the [100 200] ms window, there was 1, 6, 9, and 3 sensors in participants P2, P4, P5 and P6b, respectively, that showed a posterior probability of family $X_{\text{dyn}}^{BS}=ON$ larger than 0.75 over at least one time point. The ANOVA revealed a significant main effect of stimulus type (standard, deviant) in all sensors in all participants (*p* < 0.0001). Without correcting p-values for multiple tests performed over sensors, no significant main effect of the factor context (UC, PC) could be observed in P2, P5 and P6b (P2: *F*(1,1638) = 0.99, *p* = 0.34; P5 (larger effect across the 9 sensors): *F*(1,2163) = 3.39, *p* = 0.07; P6b (larger effect across the 3 sensors): *F*(1,3195) = 4.96, *p* = 0.03). In P4, there were 5 out 6 sensors that disclosed a significant reduction of amplitude in context PC (smaller effect across the 5 sensors: *F*(1,1987) = 8.42, p<0.004; non-significant sensor: *F*(1,1987) = 0.49, *p* = 0.48). The stimulus type by context interaction, which corresponds to the predictability effect on the MMN, was not supported by any sensors in all participants (P2: *F*(1,1638) = 0.94, *p* = 0.32; P4 (larger effect across the 6 sensors): *F*(1,1987) = 2.42, *p* = 0.12; P5 (larger effect across the 9 sensors): *F*(1,2163) = 2.23, *p* = 0.14; P6b (larger effect across the 3 sensors): *F*(1,3195) = 3.35, *p* = 0.07). The latter finding fits well with the similar difference (deviant-standard) traces obtained in contexts UC and PC represented in Figure 5C.

The single-trial modeling analysis is based on the GLM and ON/OFF family-level inference scheme employed in the *GLM analysis*. Here it was adjusted at the level of prior definition to test if model parameters depart from the values inferred in the UC context when the GLM (and nested variants) is fitted to the PC data. We restricted this analysis to the significant covariate contributions identified by the above *GLM analysis* (based on a threshold of 0.75 on posterior probability). Table 3 summarizes the resulting data point selection for each participant.

**TABLE 3:** Selection of data points for the Predictability analysis.

Subjects	Selected data points	Sensors	Time windows	Learning	Standard	Deviant	Rank	Chunk size
P2	48	7	155–320	5	22	7	21	2
P4	186	19	85–455	67	117	15	0	0
P5	81	11	115–200	69	6	6	6	0
P6b	33	7	100–215	19	8	6	1	0

*Selection was based on findings in the GLM analysis shown in Figure 6: all data points that disclosed a posterior probability larger than 0.75 in at least one regressor (except mean regressor X_0_) was included in the Predictability analysis. For each participant (rows), columns 2 to 4 provides the number of selected data points, their spatial extent (number of sensors involved) and their temporal extent (in ms). Columns 5 to 9 specify the number of selected data points that involved the corresponding regressor (multiple regressor effects could occur at the same peri-stimulus latency).*

**FIGURE 8:**
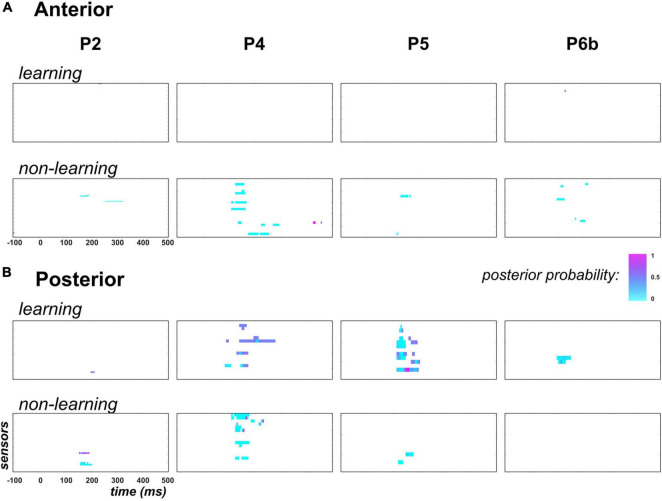
Predictability findings (family-level inference in context PC). Following the display in Figure 6, each individual map shows the posterior probability measured at each peri-stimulus time point over anterior (panel **A**) and posterior (panel **B**) temporal electrodes. In each panel, top row (learning) shows results for family τ = ON: posterior probability here indicates how likely parameter τ differs across contexts PC and UC. Bottom row (non-learning) present similar results applied to the non-learning regressor coefficients (${\text{h}}_{\text{static}}^{\text{std}}$, ${\text{h}}_{\text{static}}^{\text{dev}}$,${\text{h}}_{\text{exp}}^{\text{rank}}$ and ${\text{h}}_{\text{exp}}^{\text{cs}}$). Family-level inference for each parameter was conducted only at data points for which an effect for this parameter was found in the GLM analysis (posterior probability threshold of 0.75). For the non-learning maps, in the specific case where multiple effects could occur at the same data point in the GLM analysis, we present the largest posterior probability across these effects.

Results are presented in Figure 8.

First, regarding the predictability effect on learning, there was no data point to be tested in the anterior region, except one sensor in P2 from 230 to 235ms that here shows poor evidence for a predictability effect on τ (posterior probability lower than 0.5). Unexpectedly, in the posterior region where learning was previously found in P4, P5 and P6b, no clear evidence was observed in favor of a contextual modulation of parameter τ. Precisely, as can be seen in Figure 8B, maximum posterior probability of family τ = *ON* was equal to 0.5 in P2, 0.6 in P4, 0.9 in P5 (one electrode from 155 to 160 ms) and 0.5 in P6b.

For the static and exponential regressors, over the tested data points (columns 6 to 9, Table 3) we found low evidence supporting the modulation of their respective coefficient ($h_{*}^{*}$) by predictability. Indeed, family-level inference yielded posterior probabilities with median value over data points equal to 0.001 and 0.01 in the anterior and posterior region, respectively.

In sum, these results indicate that we here failed to reveal a predictability modulation of the MMN component and of the related perceptual learning as was evidenced at the group-level using EEG and MEG recordings (Lecaignard et al., 2021b).

## Discussion

We here presented results from single-trial ECoG data measured during the passive listening of oddball sequences with two different levels of predictability. This study had two purposes. First, to test and refine the effects that we reported in a previous study using EEG-MEG recordings. In that respect, we do reproduce an important finding by showing that a cross-trial Bayesian learning model does predict some of the inter-trial fluctuations of temporal cortex activity, at the typical latency of the scalp MMN. However, we did not observe any difference in the learning parameter between the predictable and unpredictable contexts. The second related objective was methodological and concerned the relevance of single-trial analysis for the investigation of mismatch responses. We addressed this question by evaluating the respective explanatory power of dynamic and static predictors, respectively. Therefore we combined a GLM approach with a BMR strategy. Simulations indicated a sufficient model separability given our experimental design and validated this approach. When next applied to the ECoG data, it suggested a spatial dissociation whereby the dynamic account (Bayesian learning) could be measured mostly over the posterior part of the temporal lobe and the static one over anterior electrodes. Moreover, this analysis clearly concluded in favor of a Bayesian learning explanation over an exponential one. This demonstrates the sensitivity of single-trial model fitting and strengthens the computational view of trial-by-trial fluctuations as reflecting a trajectory of precision-weighted prediction errors.

### Strengthened Evidence for Bayesian Learning During Oddball Processing

In this study, we pursued our investigation initiated with EEG-MEG recordings to shed light on perceptual learning processes and neurophysiological mechanisms during auditory oddball processing. In Stefanics et al. (2020), a similar GLM approach was employed and fitted to single-trial scalp EEG data, in the aim of investigating the repetition-suppression effect in the visual modality. Competing hypotheses were each framed as a separate GLM, that were all confronted to the data and next compared to each other using Bayesian model comparison. Here we appeal to a different methodology with a single GLM that enables mixing all hypotheses but whose respective contributions are then assessed using BMR and family-level inference. The strength of this approach is twofold. It is computationally very efficient and enables to compare many nested models. Furthermore, using a GLM approach alternative hypotheses are not strictly competing against each other in the sense that the putative most likely combination of models can be inferred given the data.

Our findings in 3 out of 4 patients present compelling evidence for Bayesian learning in posterior temporal sensors that also best show the classical MMN, between around 100 and 250 ms. Based on a posterior probability threshold of 0.9, it was measured in 5/15, 8/9, and 2/2 responsive sensors in P4, P5 and P6b, respectively. We thus succeeded in reproducing previous EEG-MEG findings, which support the view of auditory oddball processing as automatic perceptual learning (both studies involved passive listening). These results add to an emerging literature providing converging evidence from single-trial data analysis in similar experimental settings (Ostwald et al., 2012; Lieder et al., 2013; Stefanics et al., 2018; Weber et al., 2020) and more generally during sensory processing (Iglesias et al., 2013; Meyniel, 2020) where regularity learning in a context-dependent fashion is involved.

An important contribution of our study is that we succeeded in enriching this interpretation, as we here demonstrate the reliability of the explanatory power of learning dynamics (a Bayesian Surprise trajectory). This was achieved in a straightforward fashion by conducting an additional family-level inference analysis restricted to models in which the dynamic (learning) regressor was switched off. Increased evidence for the exponential regressors in this case was fairly expected, as these are the only ones that provide a time-dependent, though somewhat arbitrary, trajectory. In this way, we get closer to the model comparison performed in the study by Stefanics et al. (2020), that involved exponential, static and linear trends, but no learning model. Their study focused on the repetition-suppression effect that consists in the robust reduction of brain response amplitudes over stimulus repetition; a mechanism that is thought to participate to the MMN (Malmierca and Auksztulewicz, 2020). The authors found the exponential explanatory variable to outperform the other models. Several other studies provided similar evidence. At the neuronal level, using intracellular recordings, it was shown to account for the attenuation of the evoked discharge of visual cortical neurons (Sanchez-Vives et al., 2000). Regarding mismatch processing, plausible MMN modulations could be simulated using an exponential function, as in an attenuation model of the auditory N1 component (May and Tiitinen, 2010) or in a generative model operating at the neuronal level (Wacongne, 2016). None of these studies included a (Bayesian) learning explanatory factor. In contrast, the present analysis clearly speaks against this computational hypothesis as we measured poor evidence in favor of the rank and chunk size exponential regressors. Our findings favor such perceptual learning processes over simpler exponential accounts as the latter alternatives were clearly rejected by the data. These results fit with previous fMRI results obtained in a visual cue-association task (Iglesias et al., 2013) where a Bayesian learning model was selected over a simpler (Rescorla-Wagner) learning rule. Taken together, these findings demonstrate the informational value afforded by single-trial content, long considered as noise.

### Spatially Distinct Processes in the Temporal Cortex

Stimulus-responsive electrodes were located predominantly in the temporal region. Interestingly, the GLM analysis highlighted the spatial specificity of cognitive processes. Neurophysiological correlates of perceptual learning were located in posterior temporal electrodes whereas electrodes best distinguishing between standard and deviant stimuli, at the latency of the classical MMN, were located in the anterior part. Note that the static category was also found to correlate with posterior electrode signals, but to a far lesser extent than the dynamic one. These two functional clusters correspond to the electrode subsets that showed an MMN (Figure 5A).

The mapping of the Bayesian learning process onto posterior electrodes is in line with previous EEG-MEG findings (Lecaignard et al., 2021b). The fusion of these non-invasive observations optimized the reconstruction of the cortical generators of mismatch responses, including the MMN and an earlier component peaking at approximately 70 ms after the deviant onset (Lecaignard et al., 2021a). In the superior temporal plane, we found a bilateral contribution from the primary auditory cortex (Heschl’s gyrus), followed by a more anterior bilateral involvement of the planum polare. Bayesian learning was associated with both generators after fitting the single-trial cortical activity reconstructed at these cortical sites.

At the anterior cluster, we found large evidence for the static family (in 3/4 patients). This effect was more visible in P4 (7/11 sensors), at a latency (around 135ms) where the cortical map of the MMN displays lower amplitudes in anterior regions compared to the posterior ones. This low signal-to-noise ratio in the anterior regions may explain a greater sensitivity to the static regressor than to the dynamic one (assuming that trial-by-trial fluctuations in the case of noisy data could be well explained by the rather simple static trajectories but not by the dynamic one which in this case would be rejected as too complex). However, the fact that P6b also shows an anterior standard effect while both spatial clusters (anterior and posterior) have similar (but reverse) amplitude at the MMN speak against this hypothesis. Nevertheless, this anterior static effect contrasts with our EEG-MEG findings where, in the planum polare, Bayesian learning was found to outperform a simple ‘change detection’ model (Lecaignard et al., 2021b). Further investigations are needed to reconcile these two findings.

### Lack of Predictability Effect

We could not reproduce here our EEG-MEG findings regarding the automatic adaptation of Bayesian learning to changes in the predictability of the acoustic environment. Neither did we observe the consequence of such an adaptation onto the evoked responses (the visible tip of the iceberg; no MMN reduction was measured as reported using EEG and MEG). Furthermore and somewhat surprisingly, no modeling responsiveness was found at inferior frontal sites where MMN generators could be located in several studies (Rinne et al., 2000; Schönwiesner et al., 2007; Fulham et al., 2014; Auksztulewicz and Friston, 2015; Lecaignard et al., 2021a).

In the EEG-MEG study, adaptation of Bayesian learning was found to imply model time constant or memory span (learning parameter τ). A larger τ value was inferred from single-trial data in the predictable context. Here, since the *GLM analysis* provided strong evidence for such Bayesian learning, we expected to measure a comparable predictability modulation. Several possible explanations are discussed below as to why we did not reproduce the EEG-MEG findings.

First, the present work relies on individual analysis of data in 4 patients while the previous study relied on a group-level inference from 20 subjects. It should be noticed that individual statistical analysis of the predictability effect on the MMN component (data not shown) yielded 13 out of 20 participants showing a significantly reduced MMN in context PC (an effect measured with EEG, MEG or both). The fact that this MMN modulation was not systematically visible at the individual level (7/20 subjects did not show the effect) suggests a large inter-individual variability that could arise from the difficulty to learn the subtle predictability manipulation in passive listening (this difficulty is here even more stronger with the present paradigm, as discussed below). In this ECoG study, the implicit learning of the statistical structure of sound sequences could also be influenced by the patients’ condition.

Also, in patients P2 and P4, 39% and 26% of the trials were discarded due to artefacts (spikes and high frequency bursts of arguably muscle origin). We obtained different results in the two recording sessions acquired in P6 (P6a and P6b, separated by 1 day), and they strongly differ in their number of accepted trials (1097 and 3199, respectively). This likely speaks to the fact that single-trial data modeling requires highly informed signals to provide conclusive inference from subtle variations. This could be achieved in the EEG-MEG work by collecting a large amount of data by fusing complementary techniques (EEG, MEG) and also through a large number of participants. In the present case, although ECoG provides signals with excellent temporal and spatial resolution, individual datasets may be insufficient. Again, single-trial data analysis is a burgeoning methodology as compared to averaging methods (ERPs, oscillations) and empirical reports are therefore needed to strengthen and improve this approach.

Another aspect concerns the lack of superior frontal cortical coverage of ECoG arrays in the four participants. In our EEG-MEG study, the predictability modulation of the MMN was larger in space and time in EEG than in MEG (as can be seen in Figure 1B; Lecaignard et al., 2021b). This aspect led us assume a superior frontal generator whose radial orientation would poorly express on gradiometers (MEG is acknowledged to have a very low sensitivity to radial sources). Few studies have reported MMN generators in superior frontal cortex (Lappe et al., 2013), but we could confirm the contribution of this region to the predictability adaptation (this effect was measured over a fronto-temporal network). Here, none of the four patients presented electrodes located in those regions, and it cannot be excluded that such a predictability effect might have been observed if it had been the case.

Finally, a plausible explanation for not observing a predictability effect could be the slight change of paradigm that we implemented for this study. Indeed, here the predictable sequence was made of alternating cycles with incrementing and decrementing chunk sizes, respectively, while in our initial study, predictable sound sequences were composed of incrementing cycles only. This change was made to avoid the discontinuity at the end of each cycle (where a chunk of size 8 is followed by a chunk of size 2), which consists in a kind of rule violation (at the chunk level). However, the counterpart of this correction is a reduction of the saliency of the underlying statistical structure, making it possibly more difficult for the brain to learn implicitly and adapts accordingly. Here we are faced with the challenge inherent in investigating implicit sensory processing. Experimental manipulations should be salient enough to be processed, but subtle enough to avoid triggering explicit processing.

### Perspectives

The present analyses were based on single-trial evoked responses in the 2–20 Hz frequency band, in the aim of testing the reproducibility of and refine spatio-temporally previous EEG-MEG findings. The great informational value of ECoG is evident here, in particular through the spatial functional distinction at the MMN latency. However, the benefit of ECoG also lies in its potential to reveal fine cognitive processes from spectral analysis (Moheimanian et al., 2021; Paraskevopoulou et al., 2021). Regarding oddball processing, an ECoG study addressed the computational role of specific bandwidths from single-trial data analysis in the auditory cortex (Sedley et al., 2016). Remarkably, they could relate the gamma, beta and alpha bands to surprise, prediction updates and precision, respectively. In another study (Dürschmid et al., 2016), a predictability manipulation of deviant occurrence was also employed. Significant mismatch evoked responses in the 1–20 Hz frequency band were found in frontal and temporal electrodes but were not found to be modulated by predictability. This predictability effect was only visible in the high gamma activity, in frontal regions. Putting aside the differences in the experimental design between the two studies, the absence of predictability effect on evoked responses fits our own observations in the present work. An important next step with our data will be to explore the computational correlates of spectral responses.

## Conclusion

The original results presented here and obtained from ECoG data analysis provide further evidence for the implementation of implicit Bayesian inference processes dedicated to monitoring environmental auditory regularities. Such empirical evidence are essential in the effort to assess the computational underpinnings of perception, and to reveal the link between neurobiological mechanisms and cognitive algorithms such as predictive coding. Importantly, this study illustrates the great potential of single-trial data analysis to reveal subtle dynamic brain processes.

## Data Availability Statement

The raw data supporting the conclusions of this article will be made available by the authors, without undue reservation.

## Ethics Statement

The studies involving human participants were reviewed and approved by the Institutional Review Board of Albany Medical College; Human Research Protections Office of the United States Army Medical Research and Materiel Command. The patients/participants provided their written informed consent to participate in this study. Written informed consent was obtained from the individual(s) for the publication of any potentially identifiable images or data included in this article.

## Author Contributions

FL, AC, and JM conceptualized and designed the paradigm. PB and RB programmed the task and acquired the data. PB provided clinical information. FL and JM designed the methodology. FL implemented the computer codes, analyzed the data, and wrote the manuscript. GS, AC, PB, and JM revised the manuscript. JM, PB, and GS supervised the work. All authors contributed to the article and approved the submitted version.

## Conflict of Interest

The authors declare that the research was conducted in the absence of any commercial or financial relationships that could be construed as a potential conflict of interest.

## Publisher’s Note

All claims expressed in this article are solely those of the authors and do not necessarily represent those of their affiliated organizations, or those of the publisher, the editors and the reviewers. Any product that may be evaluated in this article, or claim that may be made by its manufacturer, is not guaranteed or endorsed by the publisher.

## Footnotes

1
https://www.bci2000.org

2
https://www.fil.ion.ucl.ac.uk/spm/

3
http://surfer.nmr.mgh.harvard.edu

4
https://bids.neuroimaging.io/
