# Supplementary material for: Dynamics of Oddball Sound Processing: Trial-by-Trial Modeling of ECoG Signals
Source: Front Hum Neurosci. 2022 Feb 10;15:794654. doi: 10.3389/fnhum.2021.794654 (PMC8866734; doi:10.3389/fnhum.2021.794654)
Supplement: Supplementary file 1 [file Data_Sheet_1.pdf]

SUPPLEMENTARY FIGURES

Dynamics of Oddball Sound Processing: Trial-by-Trial Modeling of ECoG signals

Françoise Lecaignard[1,2], Raphaëlle Bertrand[1,2], Peter Brunner[3,4,5], Anne Caclin[1,2], Gerwin Schalk[5], Jérémie Mattout[1,2]

[1] Lyon Neuroscience Research Center, CRNL; INSERM, U1028; CNRS, UMR5292; F-69000, France
[2] University Lyon 1, Lyon, F-69000, France
[3] Department of Neurosurgery, Washington University School of Medicine, St. Louis, MO, USA
[4] Department of Neurology, Albany Medical College, Albany, NY, USA
[5] National Center for Adaptive Neurotechnologies, Albany, NY, USA

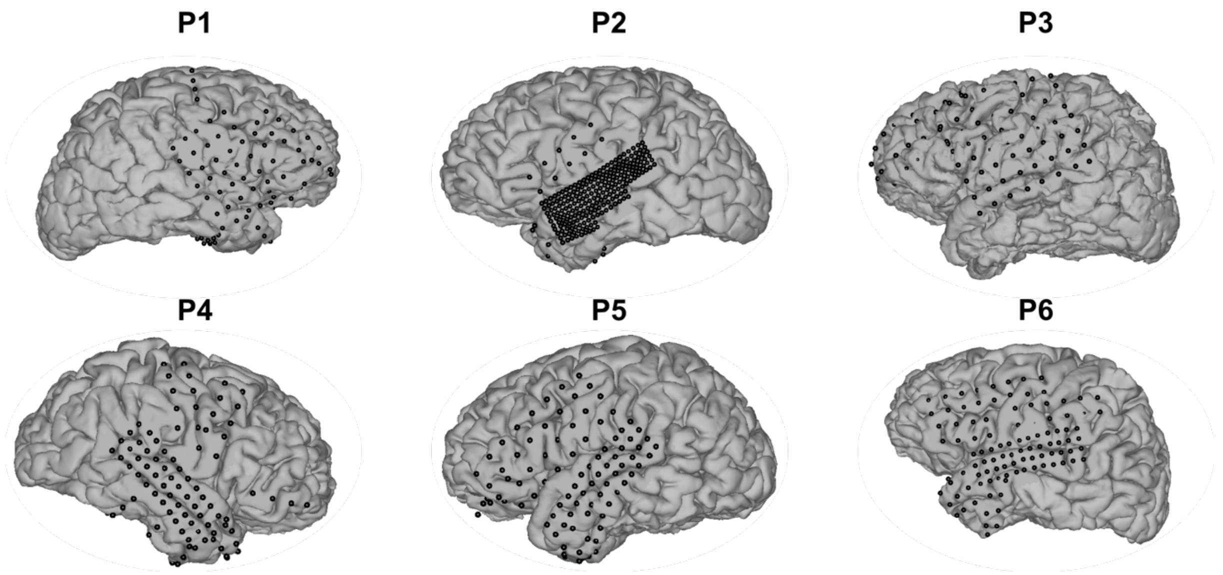

Figure S1. Localization of ECoG electrodes in all patients. Only P2, P4, P5 and P6 were included in the present findings.

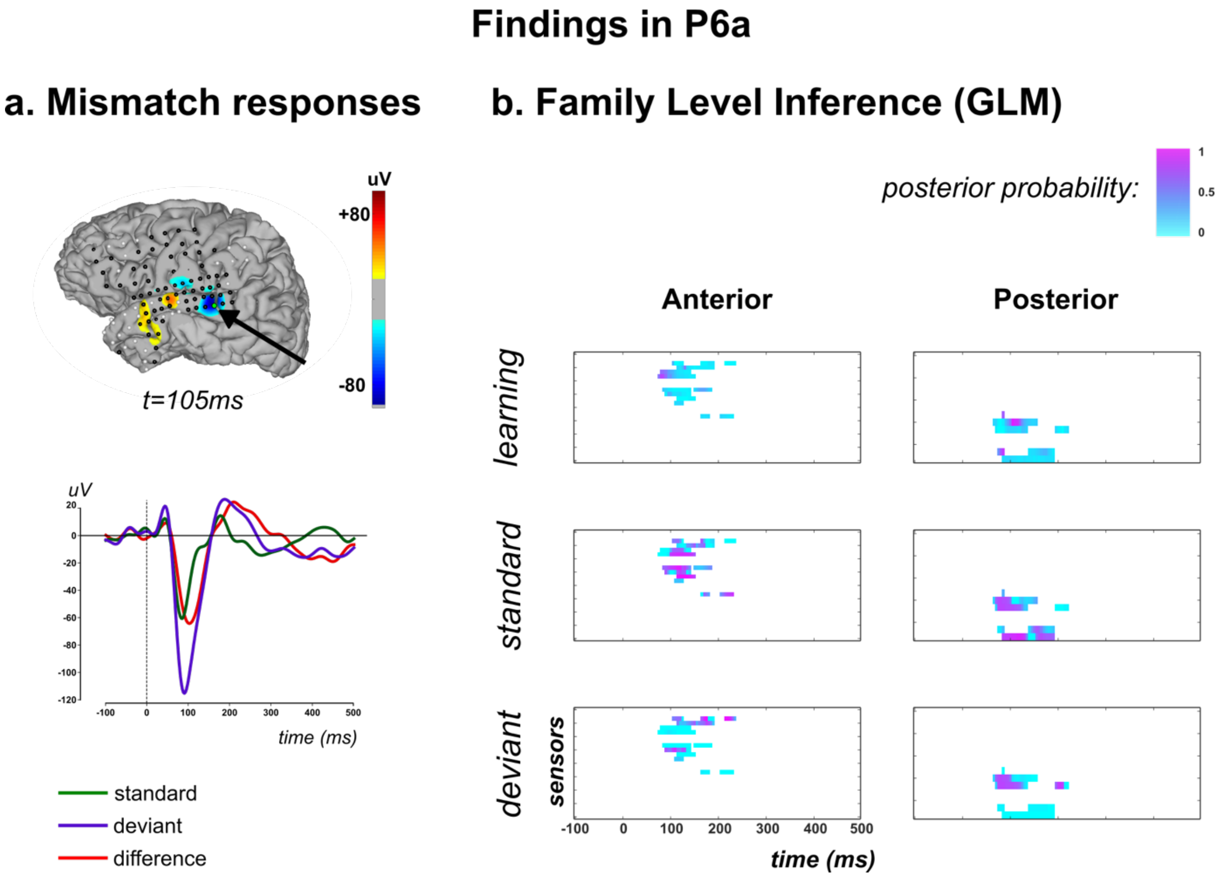

Figure S2. GLM findings in P6a. a) Evoked responses (across contexts UC and PC), using the same display as in the main text (Figure 4). b) Statistical results. Posterior probability of the learning (top row), standard (middle) and deviant (bottom) regressors at every responsive sensors and time points, over the anterior (left column) et posterior (right) temporal regions.

Findings in P3

a. Mismatch responses

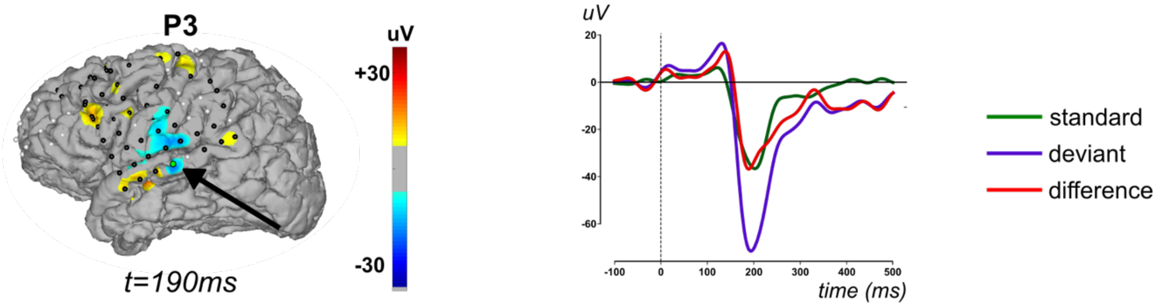

b. Family Level Inference (GLM)

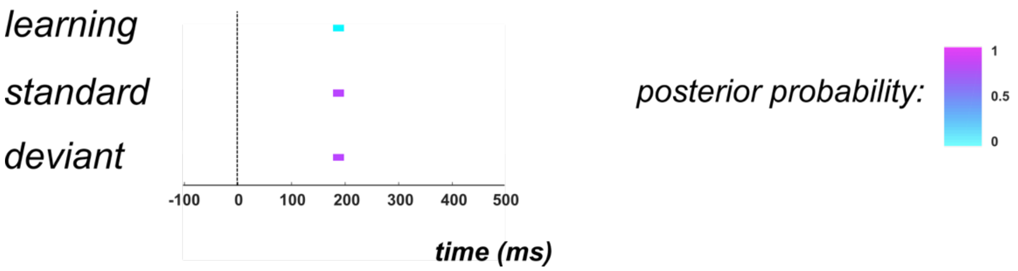

Figure S3. GLM findings in P3 (single responsive sensor). a) Evoked responses (across contexts UC and PC), using the same display as in the main text (Figure 4). b) Posterior probability measured at a single sensor (green dot in panel a) of the learning, standard and deviant regressors.

GLM study - Mean regressor coefficient (h0)

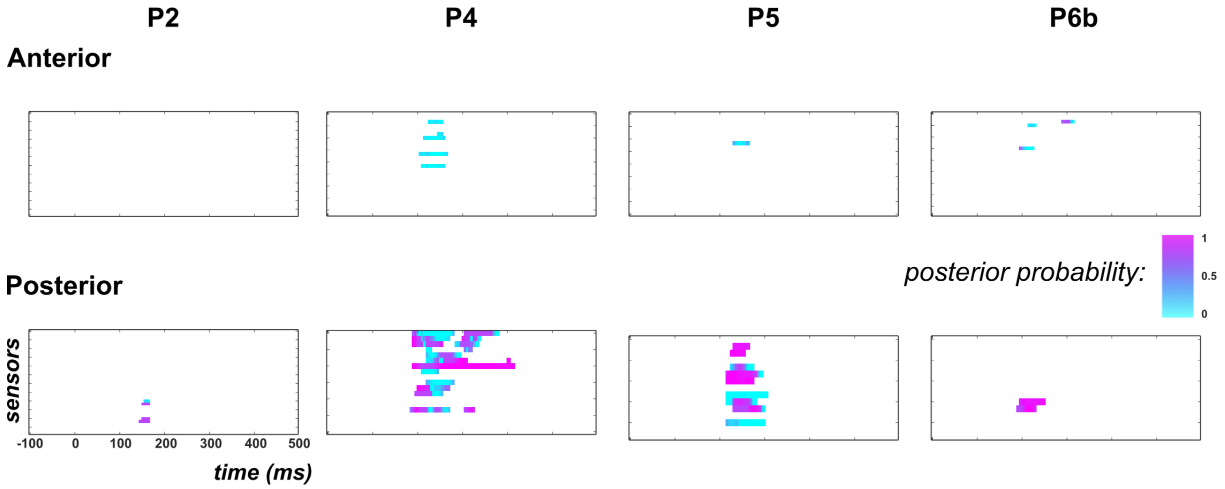

Figure S4. Family level inference for the constant regressor (GLM analysis). For each patient, spatio-temporal maps of posterior probability for family $X_O = ON$, represented using the display of Figure 5.
